# Raw meat diets are a major risk factor for carriage of third-generation cephalosporin-resistant and multidrug-resistant *E. coli* by dogs in the UK

**DOI:** 10.3389/fmicb.2024.1460143

**Published:** 2024-09-09

**Authors:** Genever Morgan, Gina Pinchbeck, Sam Haldenby, Vanessa Schmidt, Nicola Williams

**Affiliations:** ^1^Institute of Infection, Veterinary and Ecological Sciences, University of Liverpool, Neston, United Kingdom; ^2^Centre for Genomic Research, University of Liverpool, Liverpool, United Kingdom

**Keywords:** raw meat diet, dog, AMR, *E. coli*, carriage, One Health

## Abstract

**Introduction:**

Raw-meat diets (RMD) for dogs, comprising unprocessed or non-heat-treated animal material, are increasingly popular. However, RMDs have been demonstrated to be contaminated with antimicrobial resistant (AMR) bacteria, and there is concern that such diets may pose a zoonotic disease risk. Additionally, dogs fed RMD may shed more AMR- fecal bacteria compared to those fed conventional cooked diets. Data from the UK remain limited; the present study investigated the presence of AMR-*Escherichia coli* in the feces of RMD and non-RMD (NRMD)-fed dogs in the UK, the *E. coli* AMR gene complement, and the lifestyle risk factors associated with AMR- *E. coli* carriage.

**Methods:**

Fecal samples from UK-owned dogs (*N* = 193 RMD, *N* = 239 NRMD) and questionnaires discussing lifestyle factors, were obtained between October 2020-August 2021. Samples underwent culture and antimicrobial susceptibility testing to determine the presence of AMR-*E. coli*. Whole genome sequencing determined AMR gene carriage. Risk factors for the presence of AMR-*E. coli* were determined by multivariable modeling.

**Results:**

RMD dogs carried significantly more fecal AMR *E. coli* (*p* < 0.001), including third-generation cephalosporin resistant, extended-spectrum beta-lactamase (ESBL) producing, and multidrug resistant isolates and multivariable modeling confirmed raw-meat diets to be a significant risk factor. The *bla*_CTX–M–15_ gene was the most frequently identified *bla*_ESBL_ gene. The *bla*_CTX–M–55_ and *bla*_SHV–66_ genes were also prevalent and were only found in RMD dogs. The mobile colistin resistance gene, *mcr-4* was identified in one ESBL-producing *E. coli* isolate from a NRMD-fed dog.

**Conclusion:**

This study has shown that dogs fed RMD in the UK are significantly more likely to shed *E. coli* which is resistant to highest priority critically important antibiotics, and multidrug resistant *E. coli*, than dogs fed NRMD. Additionally, AMR-*E. coli* isolates from RMD-fed dogs harbor multiple, diverse, and novel AMR genes. Therefore, provision of RMD to dogs could pose an important potential threat to human and animal health, especially given the close nature of the relationship many owners share with their pets. Awareness of these findings should be shared with pet owners, veterinary and medical professionals, pet food manufacturers and public health to mitigate potential risks.

## Introduction

Raw meat diets (RMD) for pets remain a popular alternative diet choice, and while conventional cooked kibble-based diets continue to be a staple for the majority of dogs, RMD is increasingly fed as at least a constituent part of the diet for many (Dodd et al., 2020; Morgan et al., 2022; PDSA, 2022). The 2022 PDSA PAW report estimated that 7% of UK dogs were fed RMD, equating to 790,000 dogs (PDSA, 2022). RMDs are comprised of muscle, bone, skin, cartilage, tendon and organs from livestock and wild animals, which have not undergone heat treatment or cooking during the food production process (Freeman et al., 2013; Davies et al., 2019), and may be provided in a commercial pre-prepared food, or home-prepared. RMDs for dogs and cats have been demonstrated to harbor pathogenic and zoonotic organisms, including *E. coli* O157:H7, *Salmonella* spp., *Listeria monocytogenes*, *Campylobacter* spp., amongst others (Davies et al., 2019; Kaindama et al., 2020) and such bacteria have been found to be shed by dogs and cats fed RMD globally (Morley et al., 2006; Finley et al., 2007; Lefebvre et al., 2008; Leonard et al., 2015; Baede et al., 2017; Runesvärd et al., 2020; Viegas et al., 2020; Groat et al., 2022).

Furthermore, antimicrobial-resistant (AMR) bacteria have been isolated from samples of pre-prepared RMD for pets in mainland Europe (Nilsson, 2015; Baede et al., 2017; Nüesch-Inderbinen et al., 2019) and the UK (Morgan et al., 2024) and shedding of AMR bacteria by companion animals fed RMD is of increasing concern. Provision of RMD has been identified as a risk factor for canine fecal carriage of AMR *E. coli* in mainland Europe (van den Bunt et al., 2020), with greater proportions of dogs fed RMD shedding extended-spectrum beta-lactamase producing (ESBL)-*E. coli* demonstrating resistance to highest priority critically important antibiotics (HPCIAs), including third-generation cephalosporins and quinolones than those fed non-raw diets (NRMD) (Runesvärd et al., 2020).

In the UK, provision of RMD has previously been identified as a risk factor for fecal carriage of AMR *E. coli* in both healthy, non-veterinary visiting dogs (Schmidt et al., 2015) and those visiting veterinary practices (Wedley et al., 2017). Additionally, feeding RMD was identified as a risk factor for carriage of third-generation cephalosporin resistant (3GCR) *E. coli* in rural-living dogs (Sealey et al., 2022). A small study indicated that AMR, 3GCR and multidrug resistant (MDR) *E. coli* were significantly more likely to be shed by dogs fed RMD, compared to those fed NRMD, with 31% of dogs fed RMD shedding 3GCR-*E. coli*, compared to 4% of dogs fed NRMD (Groat et al., 2022).

Genes encoding 3GCR (such as *bla*_*ESBL*_ genes including those of *bla*_CTX–M_ group 1 and *bla*_CMY_) and resistance to quinolones (such as *qnr* genes), may also be co-harbored and plasmid encoded, thus mobile, increasing the potential for transmission of MDR. Plasmid-mediated AMR genes, have been identified in *E. coli* isolated from companion animals, and have been associated with those fed RMD (Groat et al., 2022; Mounsey et al., 2022; Sealey et al., 2022).

Dogs and their owners share close and frequent contact, therefore the risk posed by RMD with regards to zoonotic disease and AMR is a potential public health concern. Despite the popularity and interest surrounding RMD, data from larger scale studies surrounding the potential AMR risks associated with their provision as a diet for dogs, particularly in the UK, remain limited, and associated AMR-*E. coli* genome sequencing data is sparse.

## Aims

The aims of this study were to determine the presence of AMR *E. coli* in the feces of dogs fed either RMD or NRMD in the UK, with focus on 3GCR- *E. coli*, ESBL-producing *E. coli* and MDR-*E. coli*, alongside investigation of the AMR genes harbored by *E. coli* isolates via whole genome sequencing (WGS). Additionally, this study aimed to determine the dog and owner lifestyle risk factors for the carriage of such AMR *E. coli* in canine feces.

## Materials and methods

This study was cross-sectional in design. Data were collected between October 2020–August 2021. Participant recruitment was via email contact of dog owners who had previously participated in related studies (Morgan et al., 2022) and had agreed to be contacted further, and additionally, through social media. Following recruitment, participating households were sent a questionnaire and a fecal sample collection kit via post.

Dog owners were requested to collect one sample from a freshly evacuated stool at one time point from their dog. For multi-dog households, owners were requested to select one dog at random to participate in the study. Completed questionnaires and collected canine fecal samples were received by the laboratory by prepaid first class return post. Samples were stored at 4°C and tested within 48 h of sample delivery. Participant details were anonymised, and each sample and corresponding questionnaire was assigned a unique identification number.

The questionnaire asked about dog lifestyle and clinical factors including diet, recent antibiotic treatment, and veterinary visits, recent diarrhea, and treatment, contact with other animals and access to communal areas such as dog kennels, dog shows and public parks. It also collected data on owner factors including age, location in the country, receipt of antibiotics and place of work. Questions were multiple choice, with additional free text boxes included for owners to expand on their answers where appropriate.

Based on prior research from the UK (Groat et al., 2022), we hypothesized that the percentage of RMD-fed dogs carrying ESBL-producing *E. coli* was 30%, compared to 5% (or less) in dogs fed NRMD. A sample size of at least 32 in each group would provide 80% power to detect this difference in ESBL-producing *E. coli* with 95% confidence.

### Microbiological methods

A 1 g sample of each fecal sample was homogenized in 4 ml buffered peptone water (BPW) at room temperature and incubated aerobically at 35°C ± 1 for 18–20 h. Following incubation, a 5 μl loopful of the homogenate was inoculated onto plain chromogenic Harlequin *E. coli*/Coliform Agar (HECA) (Neogen, UK) and HECA infused with 1 μg/ml cefotaxime, a third-generation cephalosporin (HECA+Cx); all plates were incubated at 35°C ± 1 for 18–20 h. Following incubation, plates were analyzed for the presence of typical *E. coli* colonies (dark blue-violet colonies, 0.1–2 mm diameter). To explore wider AMR diversity without selection, four typical colonies, where present, were randomly picked from each HECA plate, and two colonies were picked from each HECA+Cx plate to select for cephalosporin resistant *E. coli* specifically, then individually plated onto nutrient agar (NA) (Neogen, UK) plates and incubated at 35°C ± 1 for 18–20 h.

*E. coli* isolates from plain HECA plates underwent antimicrobial susceptibility testing (AST) via the disk diffusion method. Antibiotic disks were chosen representing antimicrobials used in dogs and humans, and susceptibility tested in compliance with European Committee on Antimicrobial Susceptibility Testing (EUCAST) recommendations (EUCAST, 2022). Isolates were inoculated into sterile saline to 0.5 McFarland then a 5 μl loopful was spread onto Mueller-Hinton agar (Neogen, UK) and antibiotic disks applied. Plates were incubated aerobically at 35°C ± 1 for 18–20 h. Antimicrobials tested were ampicillin 10 μg, amoxicillin-clavulanate 20 μg/10 μg, ciprofloxacin 5 μg, tigecycline 15 μg, trimethoprim-sulphamethoxazole 1.25 μg/23.75 μg, amikacin 30 μg and meropenem 10 μg (MAST Group Ltd, Liverpool UK). A susceptible control strain of *E. coli* (ATCC 25922) was also tested.

Following incubation, zones of inhibition (ZOI) for each antibiotic disk were measured to the nearest millimeter. Human clinical breakpoints used for interpretation were as recommended by EUCAST (EUCAST, 2022) for all antibiotics other than amoxycillin-clavulanate, where the breakpoint used for interpretation was as recommended by the Clinical and Laboratory Standards Institute (CLSI, 2020). Multidrug resistance (MDR) was defined as demonstrated phenotypic resistance to three or more classes of antibiotics (Magiorakos et al., 2012).

*E. coli* isolates from HECA+Cx plates initially underwent the extended-spectrum beta-lactamase (ESBL) double-disk test using cefotaxime 5 μg, cefotaxime 5 μg +clavulanic acid 10 μg, ceftazidime 10 μg and ceftazidime 10 μg +clavulanic acid 10 μg disks (EUCAST ESBL detection set, MAST Group Ltd, Liverpool UK). Plates were incubated at 35°C ± 1 for 18–20 h. For isolates where the ZOI surrounding the cephalosporin +clavulanic acid disk was a minimum of 5 mm larger than the ZOI for the corresponding cephalosporin disk alone for ≥ 1 antibiotic pair, an ESBL-producing phenotype was confirmed, and isolates underwent full AST as described above. Non-ESBL producing third-generation cephalosporin-resistant (3GCR) *E. coli* isolates which did not demonstrate a typical positive result for ESBL production on the double disk test, but which demonstrated a pattern suggestive of AmpC production whereby there was no, or minimal, ZOI present surrounding the clavulanic acid disk(s), were also subject to full AST.

All isolates were confirmed as *E. coli* by PCR of the *usp*A gene on cell lysates (Anastasi et al., 2010). Primers used were CCGATACGCTGCCAATCAGT (forward) and ACGCAGACCGTAGGCCAGAT (reverse), with an amplicon size of 884 base pairs, and 5× HOT FIREPol^®^ Ready To Load Master Mix (Solis Biodyne, Estonia).

### Whole genome sequencing

DNA extraction was performed on ESBL-producing *E. coli* isolates using the QIAamp^®^ DNA mini kit (Qiagen, Crawley, UK).

Genomic DNA samples were submitted to the Centre for Genomic Research, University of Liverpool for Illumina NEBNext Ultra II FS DNA Library Prep, completed following the manufacturer’s protocol. Each library was quantified using Qubit and the size distribution assessed using the fragment analyser. These final libraries were pooled in equimolar amounts using the Qubit and fragment analyser data. The quantity and quality of the pool was assessed by Bioanalyzer and subsequently by qPCR using the Illumina Library Quantification Kit from Kapa (KK4854) on a Roche Light Cycler LC480II according to manufacturer’s instructions.

Following calculation of the molarity using qPCR data, template DNA was diluted to 300pM and denatured for 8 min at room temperature using freshly diluted 0.2 N sodium hydroxide (NaOH) and the reaction was subsequently terminated by the addition of 400 mM TrisCl pH = 8. To improve sequencing quality control 1% PhiX was spiked in. The libraries were sequenced on the Illumina^®^ NovaSeq 6000 platform (Illumina^®^, San Diego, USA) following the standard workflow over 1 lane of an S4 flow cell, generating 2 × 150 bp paired end reads.

Quality- and adapter-trimmed reads were assembled using SPAdes v3.13.1 (Bankevich et al., 2012). Contigs shorter than 200 bp were removed and assemblies were included in the analyses if they had (1) assembly size +/− 50% of 4.5Mb, (2) genome completeness > 90% and duplication < 10% using BUSCO v4.0.4 (Simão et al., 2015) with the gammaproteobacteria database and (3) < 10% sample read assignment to non-*Enterobacteriaceae* taxa using MetaPhlAn v2.8.1 (Segata et al., 2012). MLST profiles and allele sequences were obtained from pubmlst.org. Allele sequences were aligned to assemblies using Bowtie2 version 2.3.5.1 (Langmead and Salzberg, 2012) in sensitive mode, and best aligned alleles for each locus were selected and used to determine sequence type. Sequence types were used to infer eBURST groups, using goeBURST,^1^ where group members shared at least 2 ST locus alleles. Genes were predicted using PROKKA version 1.14.0 (Seemann, 2014) and used to reconstruct the core genome across samples, using Panaroo v1.2.4 (Tonkin-Hill et al., 2020) in “moderate” mode. Samples were filtered to only include those with fewer than 30% of core bases missing with subsequent recalculation. Phylogenetic estimation was carried out using the Panaroo core gene alignment, with IQ-TREE v2.0 (Nguyen et al., 2015), with 1,000 bootstrap replicates using the GTR model. Tree visualization was carried out at microreact.org.

AMR genes were identified by interrogating genome assemblies with RGI version 5.1.0 (Alcock et al., 2020). Plasmids were identified using PlasmidFinder v2.1.6 (Carattoli et al., 2014) and the *Enterobacteriaceae* plasmid marker database.

### Data analysis

Data analysis was undertaken in SPSS 27 [IBM Corp. (released 2020). IBM SPSS Statistics for Windows, Version 27.0. Armonk, NY: IBM Corp.]. Descriptive analysis was undertaken to determine the frequency and percentage (with 95% confidence intervals) of antimicrobial-resistant *E. coli* present at sample and isolate level for dogs fed RMD or NRMD. Comparisons between dogs fed RMD and NRMD were undertaken using the chi-square test (Fisher’s exact test for groups of *N* < 5), and significance was set at *p* < 0.05.

Descriptive analysis of categorical questionnaire response data (frequency, percentage) was undertaken. Based on the accompanying laboratory results, three outcomes were analyzed; “presence of ESBL- producing *E. coli*,” “presence of 3GCR-*E. coli*” and “presence of MDR-*E. coli*.” Odds ratios and 95% confidence intervals were generated by univariable logistic regression to identify explanatory variables associated with the three outcomes. Variables with a liberal *p*-value of < 0.3 were selected for inclusion into each multivariable model. Correlations between each variable were assessed, and where a high correlation coefficient (> 0.7) was identified, only the variable deemed most suitable was selected for inclusion into the model. Multivariable logistic regression models were built using a backward elimination method to sequentially remove variables with a *p*-value of > 0.05 until all remaining variables were significant at *p* < 0.05. Variables which had been eliminated were individually reinserted back into the model and checked to ensure that any confounding or significant variables had not been omitted. Plausible interactions between variables were also tested in the model to ensure no significant interactions had been missed and then “goodness of fit” of the final model was tested using the Hosmer-Lemeshow test.

## Results

A total of 432 (193 RMD-fed, 239 NRMD-fed) canine fecal samples were received. *E. coli* was isolated from 92.6% (400/432; 191 RMD, 209 NRMD) of samples and *E. coli* which demonstrated resistance to at least one class of antibiotics was isolated from 39.4% (76/193) of RMD-fed and 13.8% (*N* = 33/239) of NRMD-fed dogs (*p* < 0.001) (Table 1). Dogs which were fed RMD carried significantly more 3GCR-*E. coli* (*p* < 0.001), ESBL-producing *E. coli* (*p* < 0.001), multidrug-resistant (MDR) ESBL-producing *E. coli* (*p* < 0.001) and fluoroquinolone-resistant (FQR) ESBL-producing *E. coli* (*p* < 0.001) than dogs fed NRMD (Table 1).

**TABLE 1:** Sample level data [Number (*N*) and percentage (%)] describing the overall phenotypic antimicrobial resistance demonstrated by *E. coli* isolated from the feces of dogs fed either a raw (RMD, *N* = 193) or non-raw (NRMD, *N* = 239) diet.

Phenotypic resistance	Diet choice % (*N*)				*p*-value
	RMD (44.7%, *N* = 193)		NRMD (55.3%, *N* = 239)		
	*N*	% (95% CI)	*N*	% (95% CI)	
*E. coli* resistant to ≥ 1 class of antibiotics	76	39.4 (32.8–46.4)	33	13.8 (10.0–18.8)	< 0.001
Third-generation cephalosporin resistant *E. coli*	63	32.6 (26.4–39.5)	12	5.0 (2.9–8.6)	< 0.001
ESBL- producing *E. coli*	47	24.4 (18.8–30.9)	4	1.7 (0.7–4.2)	< 0.001
MDR ESBL- producing *E. coli*	32	16.6 (12.0–22.5)	3	1.3 (0.4–3.6)	< 0.001
Fluoroquinolone-resistant ESBL-producing *E. coli*	21	10.9 (7.2–16.1)	2	0.8 (0.2–3.0)	< 0.001

Of the dogs fed RMD, approximately one third shed 3GCR-*E. coli* in their feces and a quarter shed ESBL-producing *E. coli*. Additionally, 17% of RMD-fed dogs shed MDR ESBL-producing *E. coli*, compared to 1% of those fed NRMD.

Eighty-seven (*N* = 75 RMD, *N* = 12 NRMD) 3GCR *E. coli* isolates demonstrated unique resistance profiles on AST within a sample. Twenty separate resistance profiles were identified from RMD isolates, and just seven from NRMD isolates. The most frequently observed resistance profiles are presented in Table 2. The most frequently observed profile in both RMD (14.7%, *N* = 11) and NRMD (*N* = 25.0%, *N* = 3) isolates was resistance to ampicillin, amoxycillin-clavulanate, cefotaxime and ceftazidime.

**TABLE 2:** Resistance profiles of isolates submitted for whole genome sequencing (*N* = 87; RMD = 75, NRMD = 12).

Antibiotic resistance profile*	Raw (*N* = 75)	Non-Raw (*N* = 12)
	**% (*N*)**	**% (*N*)**
Amp, AmxC, Ctx, Ctz	14.7 (11)	25.0 (3)
Amp, Cip, TMS, Ctx, Ctz	10.7 (8)	8.3 (1)
Amp, Ctx, Ctz	9.3 (7)	8.3 (1)
Amp, Cip, Ctx, Ctz	6.7 (5)	16.7 (2)
Amp, Ctx	6.7 (5)	–
Amp, TMS, Ctx	6.7 (5)	8.3 (1)
Amp, TMS, Ctx, Ctz	5.3 (4)	–
Amp, Cip, TMS, Ctx	5.3 (4)	–
Amp, AmxC, Ctz	5.3 (4)	16.7 (2)
Amp, AmxC, TMS, Ctz	5.3 (4)	16.7 (2)

*Amp, ampicillin; AmxC, amoxycillin-clavulanate; Cip, ciprofloxacin; TMS, trimethoprim-sulphamethoxazole; Ctx, cefotaxime; Ctz, ceftazidime. Profiles demonstrated by more than 4 isolates for RMD are presented, with profiles represented by < 3 isolates omitted from the table. All profiles for NRMD isolates are presented.

These 87 isolates underwent whole genome sequencing (WGS) and included ESBL-producing isolates, and those which were potential pAmpC based on their cephalosporin resistance profile but were not confirmed as ESBL-producing.

Multiple and varied AMR genes were identified on WGS and full results are presented in Supplementary Appendix Table 1 with a summary presented in Table 3. The *E. coli* isolates detected from dogs fed RMD had a wider variety of AMR genes than dogs fed NRMD (Table 3). The predominant ESBL-genes in both RMD and NRMD isolates were *bla*_CTX–M_, with *bla*_CTX–M–15_ being the most frequently found (19%, 14/75 RMD; 25% 3/12 NRMD). A wide range of *bla*_CTX–M_ genes was present in RMD isolates, with 12 different genes being identified, compared to two different *bla*_CTX–M_ genes identified in NRMD isolates (*bla*_CTX–M–15_ and *bla*_CTX–M–1_). Within the RMD isolates, *bla*_CTX–M–55_ was the second-most frequently isolated ESBL-gene (12%, 9/75), however, this gene was not present in NRMD-originating isolates. Multiple *bla*_TEM_ genes were identified in the isolates, with *bla*_TEM–1_ being the most frequently isolated (Supplementary Appendix Table 1), however, in terms of ESBL-producing *bla*_TEM_ genes, *bla*_TEM–52_ and *bla*_TEM–60_ were isolated in RMD *E. coli* isolates only. Additionally, two inhibitor-resistant *bla*_TEM_ genes were identified, *bla*_TEM–78_ (*N* = 3 RMD, *N* = 1 NRMD) and *bla*_TEM–185_ (*N* = 3 RMD only). The ESBL-producing *bla*_SHV–66_ gene was only identified in RMD isolates. The ESBL *bla*_OXA–45_ gene was infrequently observed and was identified in one isolate each from RMD and NRMD-fed dogs.

**TABLE 3:** Summary table of ESBL and pAmpC genes identified in *E. coli* isolates from RMD-fed (*N* = 75 isolates) and NRMD-fed (*N* = 12 isolates) via whole genome sequencing, demonstrating percentage (%) and number (*N*) of genes present within the isolates submitted for sequencing.

Genotype		Diet choice			
		**RMD (75)**		**NRMD (12)**	
		***N***	**% (95% CI)**	***N***	**% (95% CI)**
**ESBL genes**					
*bla*_CTX–M_	CTX-M-1	5	6.7 (2.9–14.7)	1	8.3 (1.5–35.4)
	CTX-M-2	1	1.3 (0.2–7.2)	0	0
	CTX-M-9	1	1.3 (0.2–7.2)	0	0
	CTX-M-14	2	1.3 (0.2–7.2)	0	0
	CTX-M-15	14	18.7 (11.5–28.9)	3	25.0 (8.9–54.2)
	CTX-M-24	1	1.3 (0.2–7.2)	0	0
	CTX-M-27	1	1.3 (0.2–7.2)	0	0
	CTX-M-32	2	2.7 (0.7–9.2)	0	0
	CTX-M-55	9	12.0 (6.4–21.3)	0	0
	CTX-M-60	1	1.3 (0.2–7.2)	0	0
	CTX-M-65	1	1.3 (0.2–7.2)	0	0
	CTX-M-123	1	1.3 (0.2–7.2)	0	0
*bla*_TEM_	TEM-52	2	2.7 (0.7–9.2)	0	0
	TEM-60	1	1.3 (0.2–7.2)	0	0
	TEM-78*	3	4.0 (1.4–11.1)	1	8.3 (1.5–35.4)
	TEM-185*	3	4.0 (1.4–11.1)	0	0
*bla*_SHV_	SHV-66	10	(7.4–22.8)	0	0
*bla*_OXA_	OXA-45	1	1.3 (0.2–7.2)	1	8.3 (1.5–35.4)
**pAmpC genes**					
*bla*_CMY_	CMY-2	16	21.3 (13.6–31.9)	1	8.3 (1.5–35.4)
	CMY-4	1	1.3 (0.2–7.2)	0	0
	CMY-6	1	1.3 (0.2–7.2)	0	0
	CMY-44	0	0	1	8.3 (1.5–35.4)
	CMY-58	1	1.3 (0.2–7.2)	0	0
	CMY-59	1	1.3 (0.2–7.2)	0	0
	CMY-100	1	1.3 (0.2–7.2)	0	0
	CMY-132	2	2.7 (0.7–9.2)	0	0
*bla*_DHA_	DHA-1	1	1.3 (0.2–7.2)	1	8.3 (1.5–35.4)
**Quinolone resistance associated genes**					
*qnr*	*B4*	1	1.3 (0.2–7.2)	1	8.3 (1.5–35.4)
	*S1*	15	20.0 (12.5–30.4)	2	16.7 (4.7–44.8)
	*S2*	1	1.3 (0.2–7.2)	0	0
	*S7*	1	1.3 (0.2–7.2)	0	0
	*S15*	1	1.3 (0.2–7.2)	0	0
*parC*		9	12.0 (6.4–21.3)	1	8.3 (1.5–35.4)
*gyrA*		18	24.0 (15.8–34.8)	3	25.0 (8.9–54.2)
*aac(6*′*)-Ib-cr*		1	1.3 (0.2–7.2)	0	0
**Colistin resistance associated gene**					
*mcr-4*		0	0	1	8.3 (1.5–35.4)
**Rifampin resistance associated gene**					
*arr-2*		1	1.3 (0.2–7.2)	0	0
**Genes detected for other antibiotic classes**					
Tetracyclines [*tet(A)*, *tet(B)*, *tet(Y)*, *tetR*, *tetB(P)*, *tet(M)*]		40	53.3 (42.2–64.2)	4	33.3 (13.8–60.9)
Aminoglycosides*		49	65.3 (54.1–75.1)	7	58.3 (32.0–80.7)
TMS (*dfrA1*, *dfrA12*, *dfrA14*, *dfrA16*, *dfrA17*, *sul1*, *sul2*, *sul3*)		38	50.1 (39.6–61.7)	6	50.0 (25.4–70.6)
Chloramphenicol (*catl*, *cmlA6*, *cmx*)		15	20.0 (12.5–30.4)	0	0

*AAC(3)-IId, AAC(3)-IIe, AAC(6′)-Ib-cr, AAC(6′)-Ib7, AAC(6′)-Iy, ANT(2″)-Ia, ANT(3″)-IIa, ANT(4′)-IIb, APH(3″)-Ib, APH(3′)-IIa, APH(3′)-Ia, APH(6)-Ic, APH(6)-Id. *blaTEM-78 and blaTEM-185 are inhibitor-resistant genes.

Additionally, pAmpC genes were mainly observed in RMD isolates. By far the most frequently observed pAmpC gene was *bla*_CMY–2_, present in 21% (16/75) of RMD *E. coli* isolates, whereas this gene was only identified in one NRMD isolate. With regards to genes associated with plasmid-mediated quinolone resistance, five separate *qnr* genes were observed in RMD isolates, and only two in NRMD isolates. The *qnrS1* gene was most frequently isolated, Both RMD and NRMD isolates demonstrated the presence of variants of the *parC* and *gyrA* genes which mediate quinolone resistance. Concerningly, one RMD isolate carried the *aac(6*′*)-Ib-cr* gene, which can simultaneously result in resistance to fluoroquinolones with a piperazinyl group (e.g., ciprofloxacin) and aminoglycoside resistance. The mobile colistin-resistance encoding *mcr-4* gene was identified in one NRMD-originating isolate.

**FIGURE 1:**
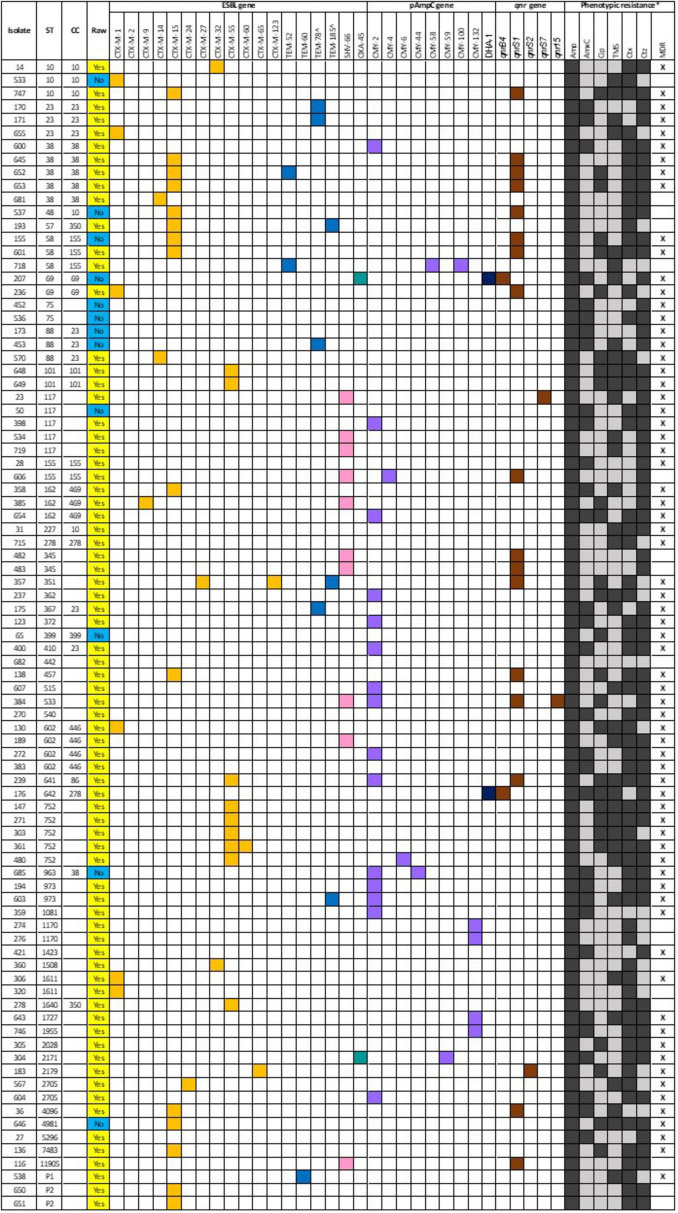
ESBL, pAmpC and quinolone resistance associated qnr genes associated with each isolate which underwent whole genome sequencing, alongside the sequence type (ST) and clonal complex (CC) identified and phenotypic resistance demonstrated via disk diffusion. For the “raw” column, a yellow box denotes a raw-fed dog isolate, whereas a blue box denotes a non-raw fed dog isolate. For the genes, a colored box indicates presence of a gene. For the phenotypic resistance, a black box denotes a resistance, and a gray box denotes susceptible. Although amikacin, tigecycline and meropenem were all tested via disk diffusion, no resistance was observed, and they have been omitted from this figure. *Amp, ampicillin; AmxC, amoxycillin-clavulanate; Cip, ciprofloxacin; TMS, trimethoprim-sulphamethoxazole; Ctx, cefotaxime; Ctz, ceftazidime; MDR, multidrug resistance. ^inhibitor-resistant genes.

**FIGURE 2:**
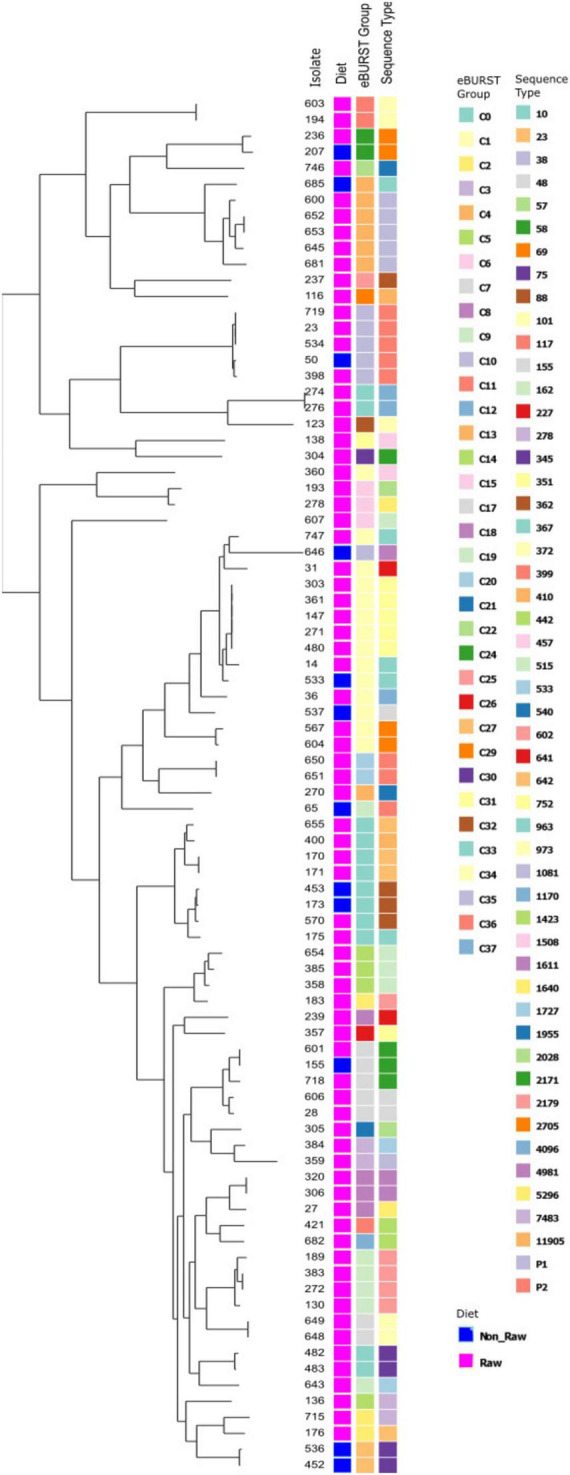
Phylogenetic tree demonstrating the eBURST groups and sequence types (ST) present, and their relationships, for *E. coli* isolates which underwent whole genome sequencing. For the “group” column, a pink box denotes raw-fed, blue denotes non-raw-fed isolate.

The ESBL, pAmpC and plasmid-mediated quinolone-resistance associated *qnr* genes present for each isolate, alongside the phenotypic AST results and the associated sequence type (ST) and clonal complex (CC) identified are presented in Figure 1. All isolates demonstrated resistance to ampicillin, and phenotypic 3GCR was indicated in all but three isolates. Ciprofloxacin resistance was demonstrated by 31% (27/87) of isolates (*N* = 24 RMD, *N* = 3 NRMD) and resistance to TMS was observed in 41% (36/87) of isolates (*N* = 32 RMD, *N* = 4 NRMD). No phenotypic resistance to tigecycline, amikacin or meropenem was identified. MDR phenotypes were present in 79% (69/87) of isolates (*N* = 59 RMD, *N* = 10 NRMD). STs, their associated *bla* genes and their phylogenetic relationships are presented in Figure 2, with an interactive phylogenetic tree available at https://microreact.org/project/jD612SJW4MUv28tQ1BmMpX-genever-v2. Fifty-two distinct *E. coli* sequence types (STs) were identified (*N* = 42 STs RMD only, *N* = 5 STs NRMD only, *N* = 5 STs both RMD and NRMD), including STs of concern such as ST10, ST38, ST58, ST69, ST155 and ST410. Two novel STs were identified across three RMD isolates. The most frequently observed STs from RMD dogs were ST38 (*N* = 5), ST117 (*N* = 4), ST602 (*N* = 4) and ST752 (*N* = 5), whereas the most common STs in isolates from NRMD dogs were ST75 (*N* = 2) and ST88 (*N* = 2).

Multiple plasmid-mediated AMR genes were often observed concurrently, particularly within RMD-isolates, however, presence of the AMR-genes was not always associated with phenotypic resistance. The presence of the *bla*_CTX–M–15_ gene was frequently associated with the presence of *qnrS1* across a range of STs. This was the case for 9 isolates (*N* = 7 RMD, *N* = 2 NRMD), and of these, 8 isolates demonstrated MDR on AST. One isolate (ST533), from a single RMD-fed dog harbored both *qnrS1* and *qnrS15*, was MDR, and demonstrated FQR and 3GCR. It was, however, not associated with the presence of *bla*_CTX–M_ genes, but *bla*SHV_–66_ and *bla*_CMY–2_ were both present. A further isolate of interest from an RMD-fed dog (ST351) carried *bla*_CTX–M–27_, *bla*_CTX–M–123_, *bla*_TEM–185_ and *qnrS1*. and was phenotypically MDR, with FQR and 3GCR. Both isolates which carried the *bla*_DHA–1_ gene (*N* = 1 RMD, *N* = 1 NRMD) also concurrently carried *qnrB4*, and were the only isolates associated with the carriage of this *qnr* gene. Both isolates demonstrated phenotypic resistance to amoxycillin-clavulanate, but only one demonstrated phenotypic FQR (RMD-fed). All ST101 and ST752 isolates harbored the *bla*_CTX–M–55_ gene. All but one of the isolates which carried *bla*_CTX–M–55_ demonstrated phenotypic MDR to combinations of ampicillin, ciprofloxacin, TMS, cefotaxime and ceftazidime. All isolates which harbored the *bla*_TEM–78_ gene demonstrated phenotypic amoxycillin-clavulanate resistance, alongside being MDR.

Multiple combinations of plasmid groups were associated with *bla*_ESBL_ gene carriage in the present study (Table 4), including IncF, IncB/O/K/Z, IncI, IncH and IncX groups. There were some associations of plasmids with specific STs; plasmid IncFII was identified in all ST752 isolates.

**TABLE 4:** Inc group plasmids associated with sequence types (STs) and ESBL genes of interest from ESBL-producing *E. coli* isolated from RMD-fed (*N* = 75 isolates) and NRMD-fed (*N* = 12 isolates) dog feces in the present study.

ESBL gene		STs associated	Plasmids associated
*bla*_CTX–M_	1	10, 23, 69, 602, 1611	IncFIA| AP001918, IncFIB(AP001918)| AP001918, IncFIC(FII)| AP001918, IncFII(pCoo)| CR942285, IncFII| AY458016, IncI1-I(gamma)| AP005147, IncY| K02380
	2	362	IncB/O/K/Z| CU928147, IncB/O/K/Z| FN868832, IncFIB(AP001918)| AP001918, IncFII(pCoo)| CR942285, IncI1-I(gamma)| AP005147
	9	278	IncI1-I(gamma)| AP005147
	14	38, 88	IncB/O/K/Z| FN868832, IncFIB(AP001918)| AP001918, IncFIC(FII)| AP001918, IncFII(pHN7A8)| JN232517, IncFII| AY458016
	15	10, 38, 48, 57, 58, 162, 457, 1170, 4981, 7843, P2	IncB/O/K/Z| CU928147, IncB/O/K/Z| FN868832, IncFIA| AP001918, IncFIB(AP001918)| AP001918, IncFIB(H89-PhagePlasmid)| HG530657, IncFIB(K)| JN233704, IncFIC(FII)| AP001918, IncFII(pCoo)| CR942285, IncFII| AY458016, IncI1-I(gamma)| AP005147, IncI2| KP347127, IncR| DQ449578, IncX1| EU370913, IncX4| FN543504
	24	2705	IncI1-I(gamma)| AP005147
	27	351	IncFIB(AP001918)| AP001918, IncFII| AY458016, IncHI2A| BX664015, IncHI2| BX664015
	32	10, 1508	IncFIB(AP001918)| AP001918, IncFII(29)| CP003035, IncHI2A| BX664015, IncHI2| BX664015, IncI2(Delta)| AP002527, IncR| DQ449578
	55	101, 641, 752, 1640	IncFIB(AP001918)| AP001918, IncFIC(FII)| AP001918, IncFII(29)| CP003035, IncFII(pHN7A8)| JN232517, IncFII(pSE11)| AP009242, IncFII| AY458016, IncHI2A| BX664015, IncHI2| BX664015, IncI1-I(gamma)| AP005147, IncX4| FN543504
	60	752	IncFIB(AP001918)| AP001918, IncFII(pSE11)| AP009242, IncFII| AY458016
	65	2179	IncFIB(AP001918)| AP001918, IncFIC(FII)| AP001918, IncI1-I(gamma)| AP005147
	123	351	IncFIB(AP001918)| AP001918, IncFII| AY458016, IncHI2A| BX664015, IncHI2| BX664015
*bla*_TEM_	52	38, 58	IncFIA| AP001918, IncFIB(AP001918)| AP001918, IncFIC(FII)| AP001918, IncI1-I(gamma)| AP005147
	78*	23, 88, 367	IncFIA| AP001918, IncFIB(AP001918)| AP001918, IncFII(pCoo)| CR942285, IncX4| FN543504, IncY| K02380
	185*	57	IncFII| AY458016
*bla*_SHV_	66	117, 155, 162, 345, 533, 602, 11905	IncB/O/K/Z| CU928147, IncB/O/K/Z| FN868832, IncFIA(HI1)| AF250878, IncFIA| AP001918, IncFIB(AP001918)| AP001918, IncFIB(H89-PhagePlasmid)| HG530657, IncFIB(K)| JN233704, IncFIC(FII)| AP001918, IncFII(pHN7A8)| JN232517, IncFII(pRSB107)| AJ851089, IncFII(pSE11)| AP009242, IncFII| AY458016, IncHI1B(pNDM-CIT)| JX182975, IncI1-I(gamma)| AP005147, IncX1| EU370913, IncX3| JN247852, IncY| K02380
*bla*_OXA_	45	69, 2171	IncFIA| AP001918, IncFIB(AP001918)| AP001918, IncFIB(pLF82-PhagePlasmid)| CU638872, IncFIC(FII)| AP001918, IncFII| AY458016, IncI2(Delta)| AP002527
*bla*_CMY_	2	38, 117, 162, 362, 372, 410, 515, 533, 602, 641, 973, 1081, 1727, 1955, 2705	IncB/O/K/Z| CU928147, IncFIB(AP001918)| AP001918, IncFIB(H89-PhagePlasmid)| HG530657, IncFIC(FII)| AP001918, IncFII(pCoo)| CR942285, IncFII(pHN7A8)| JN232517, IncFII(pRSB107)| AJ851089, IncFII(pSE11)| AP009242, IncFII| AY458016, IncHI2A| BX664015, IncHI2| BX664015, IncI1-I(gamma)| AP005147, IncI2(Delta)| AP002527, IncI2| KP347127, IncX1| EU370913, IncX3| JN247852, IncY| K02380
	4	155	IncFIB(AP001918)| AP001918, IncFII(pHN7A8)| JN232517, IncHI1B(pNDM-CIT)| JX182975, IncI1-I(gamma)| AP005147, IncX3| JN247852
	6	752	IncFIB(AP001918)| AP001918, IncFII(pSE11)| AP009242, IncFII| AY458016,
	44	963	IncFIB(AP001918)| AP001918, IncFII(29)| CP003035
	58	58	IncFIA| AP001918, IncFIB(AP001918)| AP001918, IncFIC(FII)| AP001918, IncI1-I(gamma)| AP005147
	59	2171	IncFIB(AP001918)| AP001918, IncFIB(pLF82-PhagePlasmid)| CU638872, IncFIC(FII)| AP001918, IncFII| AY458016, IncI2(Delta)| AP002527
	100	58	IncFIA| AP001918, IncFIB(AP001918)| AP001918, IncFIC(FII)| AP001918, IncI1-I(gamma)| AP005147
	132	1170	IncFIB(AP001918)| AP001918, IncFIC(FII)| AP001918, IncFII| AY458016, IncR| DQ449578
*bla*_DHA_	1	69, 642	IncFIA| AP001918, IncFIB(AP001918)| AP001918, IncFIC(FII)| AP001918, IncFII| AY458016

*Inhibitor-resistant bla_TEM_.

### Survey data analysis

A total of 432 surveys were received. Participant demographics and univariable logistic regression results are presented in Supplementary Appendix Tables 2–4. Multivariable analysis demonstrated several dog and owner lifestyle risk factors for carriage of 3GCR-*E. coli*, ESBL-producing *E. coli* and MDR *E. coli* by dogs (Table 5).

**TABLE 5:** Final multivariable regression models describing explanatory variables significantly associated with dog (*N* = 432) fecal carriage of 3GCR-, ESBL-producing and MDR-*E. coli* in the present study.

	Outcome		
**Variable**	3GCR*	ESBL*	MDR*
	**Odds ratio**	**Odds ratio**	**Odds ratio**
	**(95% CI)**	**(95% CI)**	**(95% CI)**
**Fed a raw diet**			
Yes	10.8 (4.93, 23.75)^a^	24.34 (7.09, 83.55)^a^	22.9 (5.87, 89.58)^a^
No	Ref	Ref	Ref
**Diet change last 3 months**			
Yes	–	0.24 (0.07, 0.86)^c^	0.15 (0.03, 0.77)^b^
No	–	Ref	Ref
**Types of treat fed**			
Shop bought cooked treats/biscuits			
Yes	0.5 (0.27, 0.93)^c^	0.34 (0.16, 0.72)^b^	0.41 (0.19, 0.90)^c^
No	Ref	Ref	Ref
**Dog received antibiotics in last 3 months**			
Yes	5.03 (1.84, 13.81)^b^	5.98 (1.71, 29.92)^b^	6.32 (1.84, 21.67)^b^
No	Ref	Ref	Ref
**Vet visit within last 3 months**			
Yes	–	–	2.2 (1.01, 4.80)^c^
No	–	–	Ref
**Reason for most recent vet visit**			
No visit	Ref	Ref	–
Routine	2.28 (0.94, 5.51)	2.73 (0.92, 8.07)	–
Non-emergency	0.75 (0.34, 1.62)	1.2 (0.51, 2.82)	–
Emergency	5.12 (1.31, 20.02)^c^	6.38 (1.45, 28.01)^c^	–
**Regular access to communal places**			
Dog shows			
Yes	2.6 (1.15, 5.87)^c^	–	–
No	Ref	–	–
**Dog age (years)**			
Linear	0.91 (0.84, 0.99)^c^	–	–
**Residents in house place of work**			
Nursery			
Yes	29.92 (2.06, 435.50)^b^	–	–
No	Ref	–	–
**Dog visits care homes (e.g., PAT dog)**			
Yes	–	7.11 (1.14, 44.38)^c^	–
No	–	Ref	–

*Hosmer-Lemeshow goodness of fit 3GCR: 0.471; ESBL 0.103; MDR 0.876.

*^a^p* < 0.0.01,

*^b^p* ≤ 0.01,

*^c^p* < 0.05. Ref: Reference category.

There were also some common risk factors across all three outcomes, dogs fed a raw diet and dogs which had received antibiotics in the last 3 months were significantly more likely to shed 3GCR, ESBL-producing and MDR *E. coli*. Dog owners were asked to report the type of antibiotic prescribed (if known), these descriptive results are shown in Supplementary Appendix Table 5. The most frequently prescribed antibiotic was amoxycillin-clavulanate (*N* = 19 dogs), followed by metronidazole (*N* = 7 dogs). Veterinary visits in the last 3 months were also common to all outcomes, dogs which had visited for an emergency appointment were more likely to shed 3GCR and ESBL-producing *E. coli*, whereas dogs which had attended a veterinary clinic in general were more likely to shed MDR *E. coli*. Dogs which were fed shop bought cooked treats/biscuits were less likely to shed 3GCR, ESBL-producing or MDR *E. coli*.

There were some risk factors which were unique for 3GCR and ESBL-producing *E. coli* carriage. Dogs which attended dog shows or whose owner worked in a nursery were more likely to carry 3GCR-*E. coli*, however, dogs were less likely to carry 3GCR-*E. coli* with increasing age. Dogs that visited care homes, for example “Pets as Therapy” (PAT) dogs were more likely to carry ESBL-producing *E. coli* in their feces.

## Discussion

This study specifically aimed to investigate the effect of diet on fecal AMR *E. coli* carriage by dogs and to determine the dog and owner lifestyle risk factors associated with canine carriage of AMR *E. coli*. It has provided strong evidence that provision of RMD to dogs in the UK is a significant risk factor, and that dogs fed RMD are significantly more likely to shed MDR *E. coli* and *E. coli* demonstrating resistance to HPCIAs in their feces than those fed a cooked diet.

In the present study, a quarter of dogs fed RMD carrying ESBL-producing *E. coli*, and approximately one third of dogs fed RMD carrying 3GCR-*E. coli*. The provision of raw meat to dogs has been identified as a risk factor for AMR *E. coli* carriage globally (Lefebvre et al., 2008; Baede et al., 2015; Leonard et al., 2015; Runesvärd et al., 2020; van den Bunt et al., 2020) as well as in previous smaller studies in the UK (Wedley et al., 2017; Sealey et al., 2022). Additionally, small studies in Sweden and the UK have also observed significantly greater 3GCR- and ESBL-producing *E. coli* carriage in dogs fed RMD than those fed NRMD (Runesvärd et al., 2020; Groat et al., 2022). The findings of the present study support those of previous studies, albeit on a larger scale, highlighting the concerning prevalence of *E. coli* demonstrating resistance to HPCIAs in the feces of dogs fed RMD in the UK, and providing further detail surrounding the phenotypic AMR profiles of *E. coli* carried by RMD-fed dogs, and the associated genotypes.

There was a significantly greater prevalence of phenotypic resistance to ampicillin, amoxycillin-clavulanate, TMS and ciprofloxacin in the AMR-*E. coli* isolated from dogs fed RMD than NRMD. High levels of phenotypic resistance to ampicillin, amoxycillin-clavulanate and/or TMS have been reported in dogs fed RMD previously in the UK and Sweden (Schmidt et al., 2015; Runesvärd et al., 2020; Groat et al., 2022), and a UK study of 16-week-old puppies identified that provision of a raw diet was the most substantial risk factor for FQR *E. coli* carriage (Mounsey et al., 2022). The findings of the present study are interesting as two previous studies demonstrated no (Groat et al., 2022) or uncommon (Schmidt et al., 2015) phenotypic fluoroquinolone resistance in *E. coli* isolated from healthy adult dogs in the UK. However, in the present study approximately 11% of RMD-fed dogs carried ciprofloxacin-resistant ESBL-producing *E. coli* (compared to < 1% of NRMD-fed dogs), and this was frequently associated with MDR. No carbapenem resistance was demonstrated in dogs fed either diet in the present study, a finding which echoes that of Runesvärd et al. (2020).

In addition, isolates from RMD-fed dogs demonstrated more varied STs and a great diversity of ESBL genes. Furthermore, STs observed in *E. coli* isolated from RMD-fed dogs were similar those present in samples of UK RMDs (Morgan et al., 2024), and included globally disseminated uropathogenic STs 58 and 69; ST155, which has major importance in the plasmid mediated spread of ESBL-genes from animals to humans (Matamoros et al., 2017); and ST602, which is commonly isolated from UK livestock (Ludden et al., 2019). The most frequently observed genes in *E. coli* isolates from RMD-fed dogs in the present study were *bla*_CTX–M–15_, *bla*_CTX–M–55_ and *bla*_SHV–66_. While *bla*_CTX–M–15_ was identified, albeit far less frequently, in dogs fed NRMD, no *bla*_CTX–M–55_ or *bla*_SHV–66_ was present in isolates from NRMD-fed dogs. The presence of *bla*_CTX–M–15_ was frequently associated with concurrent *qnrS1* carriage, as well as MDR, and was present across a range of STs. There are few studies which have specifically investigated the resistance genes present in *E. coli* isolated from dogs fed raw diets. However, *bla*_CTX–M–15_ has been identified as the most prevalent *bla*_ESBL_ gene in samples of UK raw pet food (Morgan et al., 2024), and has been isolated from poultry and pigs at slaughter in the UK (Randall et al., 2011; Veterinary Medicines Directorate, 2021, 2022) While previous studies have demonstrated a predominance of *bla*_CTX–M–1_ in the UK healthy dog population (Wedley et al., 2017; Mounsey et al., 2022), this gene was only observed in 5 isolates from RMD-fed and one isolate from NRMD-fed dogs in the present study. The *bla*_CTX–M–15_ gene is the most frequently isolated *bla*_CTX–M_ gene in canine *E. coli* in other countries including the USA (Lv et al., 2013), Canada (Cormier et al., 2019) and Portugal (Carvalho et al., 2021). It is also the most commonly identified *bla*ESBL gene associated with human *E. coli* infections in the UK (Woodford, 2008; Woodford et al., 2011). The dominance of *bla*_CTX–M–15_ across a range of STs in the present study is interesting and, along with the WGS findings from other studies (Timofte et al., 2016; Singleton et al., 2021; Sealey et al., 2022), may demonstrate an increase in this particular gene within the canine population in the UK in general, alongside a decrease in *bla*_CTX–M–1_ carriage, as well as potentially an increased risk of *bla*_CTX–M–15_ carriage in RMD-fed dogs. A recent study of canine fecal *E. coli* from dogs in the South West of England demonstrated a predominance of the *bla*_CTX–M–15_ gene in urban dogs, but not rural dogs, however, excretion of *E. coli* with *bla*_CTX–M_ genes was significantly associated with RMD-feeding in both urban and rural dogs (Sealey et al., 2022).

The *bla*_CTX–M–55_ gene is derived from *bla*_CTX–M–15_ (He et al., 2015) and is frequently identified in humans, as well as being reported in food-producing animals and pets, in China (Sun et al., 2010; Lv et al., 2013; Zhang et al., 2014). However, *bla*_CTX–M–55_ is infrequently identified in dogs in other countries, and has been reported in low numbers previously in studies from Korea (Tamang et al., 2012), Canada (Cormier et al., 2019), Portugal (Carvalho et al., 2021), France (Lupo et al., 2018), Switzerland (Zogg et al., 2018) and the Netherlands (Baede et al., 2015). Interestingly, a study of dogs fed either RMD or a conventional cooked diet in Brazil found that ESBL-producing *E. coli* was only shed by RMD-fed dogs, and the most commonly identified *bla*ESBL gene was *bla*_CTX–M–55_ (Ramos et al., 2022). The high prevalence of *bla*_CTX–M–55_ in the present study (12% of RMD isolates) is a particularly intriguing finding; it has only been reported once before in dogs in the UK, in *E. coli* isolates from clinical samples (Bortolami et al., 2019). All isolates which carried *bla*_CTX–M–55_ in this study except one demonstrated MDR. *bla*_CTX–M–55_ has been identified in healthy pigs at slaughter in the UK (Veterinary Medicines Directorate, 2022), and was the most frequently identified *bla*_ESBL_ gene in healthy broilers (Veterinary Medicines Directorate, 2021). It has also been identified in pre-prepared RMD containing duck in the UK (Morgan et al., 2024). Therefore *bla*_CTX–M–55_ could be an emerging *bla*_ESBL_ gene of interest within Europe, as well as within the UK dog population and may be associated with provision of raw meat, particularly poultry.

The identification of *bla*_SHV–66_ in *E. coli* isolated from dogs fed RMD, which was not present in *E. coli* isolated from NRMD-fed dogs, is also of relevance. *bla*_SHV–66_ is usually more frequently associated with *Klebsiella* spp (Shibu et al., 2021), although has been reported in equine clinical *E. coli* isolates from one study in the UK (Isgren, 2020). Other ESBL-producing *bla*_SHV_ genes, in particular *bla*_SHV–12_, have been associated with *E. coli* isolated from dogs in Spain, Switzerland and France (Alonso et al., 2017; Zogg et al., 2018; Dupouy et al., 2019). Two studies in the UK have identified *bla*_SHV–12_ carriage in canine *E. coli* from single dogs (Singleton et al., 2021; Sealey et al., 2022), however, other UK studies did not isolate any *bla*_SHV_ genes from canine fecal *E. coli* (Wedley et al., 2017; Schmidt et al., 2018; Groat et al., 2022; Mounsey et al., 2022). This is the first report of *bla*_SHV–66_ presence in ESBL-producing *E. coli* isolated from dogs which may suggest that *bla*_SHV–66_ is an emerging *bla*_ESBL_ gene of concern.

It is unsurprising that the most prevalent pAmpC gene in this study was *bla*_CMY–2_, present across a range of STs, as this is the most frequently isolated pAmpC gene from *E. coli* of animal and human origin (Denisuik et al., 2013; Hansen et al., 2016). Additionally, *bla*_CMY–2_ has been demonstrated in *E. coli* isolated from raw pet food in the UK and mainland Europe (Nilsson, 2015; Baede et al., 2017; Morgan et al., 2024). Dogs have been frequently shown to carry *E. coli* which harbors *bla*_CMY–2_ in previous studies from South Korea, the Netherlands, Denmark, Costa Rica, France and the UK (Tamang et al., 2012; Baede et al., 2015; Hansen et al., 2016; Rodríguez-González et al., 2020; Haenni et al., 2022; Sealey et al., 2022). However, although it was isolated from *E. coli* from one NRMD-fed dog in the present study, far more *E. coli* isolates from RMD-fed dogs were demonstrated to carry this gene, suggesting that provision of RMD could be a risk for *bla*_CMY–2_ carriage. This finding is also supported by the multivariable model results demonstrating provision of RMD to be a risk factor for phenotypic 3GCR-*E. coli* carriage by dogs.

Of concern was the identification of the *mcr-4* (ST4981, isolated from a NRMD-fed dog) gene in this study, which confers plasmid-mediated resistance to colistin. This isolate was also phenotypically MDR. The *mcr-4* gene has previously been reported in *K. pneumoniae* isolated from canine feces in China (Wang et al., 2021), however, to the author’s knowledge, this is the first report of isolation of this gene from canine *E. coli*.

Although the greatest odds for AMR *E. coli* shedding by dogs was associated with RMD provision, there were additional risk factors identified across the three models tested (ESBL-producing *E. coli*, 3GCR-*E. coli* and MDR-*E. coli*). The provision of antibiotics in the last 3 months was a significant risk factor, and has been identified as a risk factor for carriage of AMR *E. coli* by dogs over this timeframe previously (Gandolfi-Decristophoris et al., 2013; Wedley et al., 2017). Treatment with specific antibiotics has been linked with AMR *E. coli* carriage in dogs; the provision of oral cephalexin has been associated with selection of *bla*_CMY–2_ producing *E. coli* (Damborg et al., 2011), and carriage of MDR *E. coli* has been attributed to the use of fluoroquinolones (Gibson et al., 2011; Leite-Martins et al., 2014), amoxycillin-clavulanate and cefovecin (Schmidt et al., 2018). Fluoroquinolone use was not widely reported in the present study, with amoxycillin-clavulanate being the most frequently prescribed antibiotic reported.

Visiting a veterinary practice in the last 3 months was a further risk factor for AMR *E. coli* carriage, with an emergency visit specifically being significant for ESBL-producing and 3GCR *E. coli*. Previous studies have identified veterinary hospitals as sources of ESBL-producing *E. coli* (Timofte et al., 2016; Schmitt et al., 2021), with carriage by staff (Royden et al., 2019) and patients being reported. A further study identified frequent carriage of AMR *E. coli* by vet-visiting dogs, with resistance to ampicillin, tetracycline and trimethoprim most commonly detected (Wedley et al., 2017). As opposed to previous studies where hospitalization and length of stay was a significant risk factor for MDR *E. coli* (Gibson et al., 2011; Tuerena et al., 2016; Haenni et al., 2022), hospitalization was not significant for any of the AMR outcomes in the present study.

Research is required to investigate the potential for transmission and co-carriage of AMR *E. coli* between dogs, in-contact people, and the environment. Dogs and their owners frequently share close contact, especially within the home where behaviors such as sharing of soft furnishings and beds, dogs sitting on the owners lap, and dogs licking owners hands and faces occur (Westgarth et al., 2008), as well as owners kissing their pets (do Vale et al., 2021). It is this close relationship, and the behaviors associated with it, which may pose a particularly high risk for transmission of AMR-bacteria between pets and their owners. In particular, risky behaviors around food such as sharing plates, utensils and allowing pets to eat from bare hands is reported, despite owners potentially being aware of the zoonotic disease potential (Dickson et al., 2019). Dogs and humans in close contact, either within the home or within another close-contact environment such as a shelter or veterinary hospital environment, have been demonstrated to share AMR *E. coli* with similar resistance genes and resistance patterns (Sidjabat et al., 2006; Toombs-Ruane et al., 2020; Cozma et al., 2022; Naziri et al., 2022), and AMR *E. coli* of the same sequence type (Johnson et al., 2016; Grönthal et al., 2018). ESBL and AmpC-producing *E. coli* of the same strain has been identified between human patients with urinary tract infections and pet dogs in the same household, suggesting within-household transmission does occur (Johnson et al., 2016; Toombs-Ruane et al., 2020).

The findings of the present study highlight that such close contact should be of particular concern with dogs fed a raw meat diet, where the potential for contact with foodborne zoonotic pathogens is greater. Few studies have investigated the risks of transmission and co-carriage of AMR *E. coli* within a pet-owning household in relation to provision of a raw diet specifically. A study from the UK identified a common *E. coli* lineage (ST744) carried by a raw fed puppy and isolated from a human urinary tract infection within a local area (Mounsey et al., 2022). A previous study from The Netherlands identified co-carriage of ESBL-producing *E. coli* between dogs and their owners in a small number of households, and observed that provision of RMD was a risk factor for ESBL-producing *E. coli* carriage in dogs (van den Bunt et al., 2020). Carriage of AMR *E. coli* of STs which are known to be of clinical importance in human medicine has been identified in the present study to a greater degree in dogs fed RMD, associated with mobile transmissible genetic elements. Therefore, it stands to reason that dogs fed RMD could pose an increased public health risk for transmission of AMR *E. coli*, however, further research is required to investigate this risk.

This study relied on direct contact using email of dog owners who had previously volunteered to take part in related studies, and via social media, thus there may have been an element of bias; certain owner demographics (such as those without access to social media), and populations of dogs where raw feeding may be regularly utilized (such as hunt kennels) may be underrepresented. Owner responses within the survey could be subject to recall bias, however, this is unlikely to affected recall around the overall food type.

The HECA media used for bacterial isolation allowed easy recognition of *E. coli* colonies. However, it is possible some colonies could be missed if there was a slight deviation from the expected color for any reason. This could lead to underestimation of *E. coli* presence at sample level. A set number of *E. coli* picks were taken from each agar plate. This method aims to obtain a representative sample by sampling multiple colonies at random, however, does mean that there could be an over- or underrepresentation of the level of AMR present, depending on the colonies picked. Finally, the presence of the AMR-genes identified by WGS in this study was not always associated with phenotypic resistance; interpretation of the AMR genes must be undertaken with caution as their presence does not necessarily indicate that resistance will be demonstrated. Further research is needed to determine the transmissibility of genes, however, the identification of these plasmid-mediated genes should be cause for concern surrounding the potential for spread of genes capable of mediating resistance to HPCIAs to other bacteria.

## Conclusion

This study has contributed to the growing body of evidence to suggest that provision of RMD to dogs is a public health concern. Dogs fed RMD were demonstrated to shed significantly greater proportions of *E. coli* resistant to HPCIAs than dogs fed NRMD. STs and ESBL genes were identified which are linked to those identified in livestock and humans, and associated with clinical disease in both humans and animals, and novel AMR genes not previously identified in healthy dogs were detected. This constitutes a potential One Health concern, as well as a concern for animal health and welfare. Further research is required to investigate the risks of co-carriage and transmission of AMR *E. coli* with respect to dogs, their owners and their environment, nevertheless, provision of RMD as a pet food choice should be considered with caution and efforts should be made to continue to engage with pet owners, pet food retailers, veterinary and medical professional with regards to the AMR bacteria risks associated with RMD feeding.

## Data Availability

The study questionnaire is available on request. The whole genome sequencing data presented in the study are deposited in the ENA repository, accession number PRJEB77569.

## Footnotes

1
https://github.com/jacarrico/goeburst

## References

[B1] Alcock B. Raphenya A. Lau T. Tsang K. Bouchard M. Edalatmand A. (2020). CARD 2020: Antibiotic resistome surveillance with the comprehensive antibiotic resistance database. *Nucleic Acids Res.* 48 D517–D525. 10.1093/nar/gkz935 31665441 PMC7145624

[B2] Alonso C. Michael G. Li J. Somalo S. Simón C. Wang Y. (2017). Analysis of blaSHV-12-carrying *Escherichia coli* clones and plasmids from human, animal and food sources. *J. Antimicrob. Chemother.* 72 1589–1596. 10.1093/jac/dkx024 28333184

[B3] Anastasi E. Matthews B. Gundogdu A. Vollmerhausen T. Ramos N. Stratton H. (2010). Prevalence and persistence of *Escherichia coli* strains with uropathogenic virulence characteristics in sewage treatment plants. *Appl. Environ. Microbiol.* 76 5882–5886. 10.1128/AEM.00141-10 20622128 PMC2935044

[B4] Baede V. Broens E. Spaninks M. Timmerman A. Graveland H. Wagenaar J. (2017). Raw pet food as a risk factor for shedding of extended-spectrum beta-lactamase-producing *Enterobacteriaceae* in household cats. *PLoS One* 12:e0187239. 10.1371/journal.pone.0187239 29095871 PMC5667807

[B5] Baede V. Wagenaar J. Broens E. Duim B. Dohmen W. Nijsse R. (2015). Longitudinal study of extended-spectrum-β-lactamase- and AmpC-producing *Enterobacteriaceae* in household dogs. *Antimicrob. Agents Chemother.* 59 3117–3124. 10.1128/AAC.04576-14 25779568 PMC4432141

[B6] Bankevich A. Nurk S. Antipov D. Gurevich A. Dvorkin M. Kulikov A. (2012). SPAdes: A new genome assembly algorithm and its applications to single-cell sequencing. *J. Comput. Biol.* 19 455–477. 10.1089/cmb.2012.0021 22506599 PMC3342519

[B7] Bortolami A. Zendri F. Maciuca E. Wattret A. Ellis C. Schmidt V. (2019). Diversity, virulence, and clinical significance of extended-spectrum β-lactamase- and pAmpC-producing *Escherichia coli* from companion animals. *Front. Microbiol.* 10:1260. 10.3389/fmicb.2019.01260 31231344 PMC6560200

[B8] Carattoli A. Zankari E. García-Fernández A. Voldby Larsen M. Lund O. Villa L. (2014). In silico detection and typing of plasmids using PlasmidFinder and plasmid multilocus sequence typing. *Antimicrob. Agents Chemother.* 58 3895–3903. 10.1128/AAC.02412-14 24777092 PMC4068535

[B9] Carvalho I. Cunha R. Martins C. Martínez-Álvarez S. Safia Chenouf N. Pimenta P. (2021). Antimicrobial resistance genes and diversity of clones among faecal ESBL-producing *Escherichia coli* isolated from healthy and sick dogs living in portugal. *Antibiotics (Basel)* 10:1013. 10.3390/antibiotics10081013 34439063 PMC8388948

[B10] CLSI (2020). *CLSI M100-ED29: 2021 Performance standards for antimicrobial susceptibility testing*, 30th Edn. Wayne, PA: CLSI.

[B11] Cormier A. Zhang P. Chalmers G. Weese J. Deckert A. Mulvey M. (2019). Diversity of CTX-M-positive *Escherichia coli* recovered from animals in Canada. *Vet. Microbiol.* 231 71–75. 10.1016/j.vetmic.2019.02.031 30955827

[B12] Cozma A. Rimbu C. Zendri F. Maciuca I. Timofte D. (2022). Clonal dissemination of extended-spectrum cephalosporin-resistant enterobacterales between dogs and humans in households and animal shelters of Romania. *Antibiotics (Basel)* 11:1242. 10.3390/antibiotics11091242 36140020 PMC9495119

[B13] Damborg P. Gaustad I. Olsen J. Guardabassi L. (2011). Selection of CMY-2 producing *Escherichia coli* in the faecal flora of dogs treated with cephalexin. *Vet. Microbiol.* 151 404–408. 10.1016/j.vetmic.2011.03.015 21497459

[B14] Davies R. Lawes J. Wales A. (2019). Raw diets for dogs and cats: A review, with particular reference to microbiological hazards. *J. Small Anim. Pract.* 60 329–339. 10.1111/jsap.13000 31025713 PMC6849757

[B15] Denisuik A. Lagacé-Wiens P. Pitout J. Mulvey M. Simner P. Tailor F. (2013). Molecular epidemiology of extended-spectrum β- lactamase-, AmpC β-lactamase- and carbapenemase-producing *Escherichia coli* and *Klebsiella pneumoniae* isolated from Canadian hospitals over a 5 year period: CANWARD 2007-11. *J. Antimicrob. Chemother.* 68 i57–i65. 10.1093/jac/dkt027 23587779

[B16] Dickson A. Smith M. Smith F. Park J. King C. Currie K. (2019). Understanding the relationship between pet owners and their companion animals as a key context for antimicrobial resistance-related behaviours: An interpretative phenomenological analysis. *Health Psychol. Behav. Med.* 7 45–61. 10.1080/21642850.2019.1577738 34040838 PMC8114347

[B17] do Vale B. Lopes A. Fontes M. Silvestre M. Cardoso L. Coelho A. C. (2021). A cross-sectional study of knowledge on ownership, zoonoses and practices among pet owners in Northern Portugal. *Animals (Basel)* 11:3543. 10.3390/ani11123543 34944317 PMC8697889

[B18] Dodd S. Cave N. Abood S. Shoveller A. Adolphe J. Verbrugghe A. (2020). An observational study of pet feeding practices and how these have changed between 2008 and 2018. *Vet. Rec.* 186 643. 10.1136/vr.105828 32554799

[B19] Dupouy V. Abdelli M. Moyano G. Arpaillange N. Bibbal D. Cadiergues M. (2019). Prevalence of beta-lactam and quinolone/fluoroquinolone resistance in *Enterobacteriaceae* from dogs in france and spain-characterization of ESBL/PAMPC isolates, genes, and conjugative plasmids. *Front. Vet. Sci.* 6:279. 10.3389/fvets.2019.00279 31544108 PMC6730528

[B20] EUCAST (2022). *Breakpoint tables for interpretation of MICs and zone diameters. Version 12.0, 2022.* Växjö: The European Committee on Antimicrobial Susceptibility Testing.

[B21] Finley R. Ribble C. Aramini J. Vandermeer M. Popa M. Litman M. (2007). The risk of *Salmonellae* shedding by dogs fed *Salmonella*-contaminated commercial raw food diets. *Can. Vet. J.* 48 69–75. 17310625 PMC1716752

[B22] Freeman L. Chandler M. Hamper B. Weeth L. (2013). Current knowledge about the risks and benefits of raw meat-based diets for dogs and cats. *J. Am. Vet. Med. Assoc.* 243 1549–1558. 10.2460/javma.243.11.1549 24261804

[B23] Gandolfi-Decristophoris P. Petrini O. Ruggeri-Bernardi N. Schelling E. (2013). Extended-spectrum β-lactamase-producing *Enterobacteriaceae* in healthy companion animals living in nursing homes and in the community. *Am. J. Infect. Control* 41 831–835. 10.1016/j.ajic.2012.11.013 23422230

[B24] Gibson J. Morton J. Cobbold R. Filippich L. Trott D. (2011). Risk factors for multidrug-resistant *Escherichia coli* rectal colonization of dogs on admission to a veterinary hospital. *Epidemiol. Infect.* 139 197–205. 10.1017/S0950268810000798 20392305

[B25] Groat E. Williams N. Pinchbeck G. Warner B. Simpson A. Schmidt V. M. (2022). UK dogs eating raw meat diets have higher risk of *Salmonella* and antimicrobial-resistant *Escherichia coli* faecal carriage. *J. Small Anim. Pract.* 63 435–441. 10.1111/jsap.13488 35191029 PMC9305152

[B26] Grönthal T. Österblad M. Eklund M. Jalava J. Nykäsenoja S. Pekkanen K. (2018). Sharing more than friendship - transmission of NDM-5 ST167 and CTX-M-9 ST69 *Escherichia coli* between dogs and humans in a family, Finland, 2015. *Euro Surveill.* 23:1700497. 10.2807/1560-7917.ES.2018.23.27.1700497 29991384 PMC6152158

[B27] Haenni M. Boulouis H. Lagrée A. Drapeau A. Va F. Billet M. (2022). Enterobacterales high-risk clones and plasmids spreading blaESBL/AmpC and blaOXA-48 genes within and between hospitalized dogs and their environment. *J. Antimicrob. Chemother.* 77 2754–2762. 10.1093/jac/dkac268 35983589

[B28] Hansen K. Bortolaia V. Nielsen C. Nielsen J. Schønning K. Agersø Y. (2016). Host-specific patterns of genetic diversity among IncI1-Iγ and IncK plasmids encoding CMY-2 β-lactamase in *Escherichia coli* isolates from humans, poultry meat, poultry, and dogs in Denmark. *Appl. Environ. Microbiol.* 82 4705–4714. 10.1128/AEM.00495-16 27235431 PMC4984282

[B29] He D. Chiou J. Zeng Z. Liu L. Chen X. Zeng L. (2015). Residues distal to the active site contribute to enhanced catalytic activity of variant and hybrid β-lactamases derived from CTX-M-14 and CTX-M-15. *Antimicrob. Agents Chemother.* 59 5976–5983. 10.1128/AAC.04920-14 26169409 PMC4576060

[B30] Isgren C. M. (2020). *The emerging problem of antimicrobial resistance in horses: Investigating faecal carriage and environmental contamination with resistant Escherichia coli* in equine hospitals and clinical infections with multidrug resistant bacteria, [Ph.D. thesis]. Liverpool: University of Liverpool.

[B31] Johnson J. Davis G. Clabots C. Johnston B. Porter S. DebRoy C. (2016). Household clustering of *Escherichia coli* sequence type 131 clinical and fecal isolates according to whole genome sequence analysis. *Open Forum Infect. Dis.* 3:ofw129. 10.1093/ofid/ofw129 27703993 PMC5047392

[B32] Kaindama L. Jenkins C. Aird H. Jorgensen F. Stoker K. Byrne L. (2020). A cluster of Shiga Toxin-producing *Escherichia coli* O157:H7 highlights raw pet food as an emerging potential source of infection in humans. *Epidemiol. Infect.* 149:e124. 10.1017/S0950268821001072 33955833 PMC8161292

[B33] Langmead B. Salzberg S. (2012). Fast gapped-read alignment with Bowtie 2. *Nat. Methods* 9 357–359. 10.1038/nmeth.1923 22388286 PMC3322381

[B34] Lefebvre S. Reid-Smith R. Boerlin P. Weese J. (2008). Evaluation of the risks of shedding *Salmonella*e and other potential pathogens by therapy dogs fed raw diets in Ontario and Alberta. *Zoonoses Public Health* 55 470–480. 10.1111/j.1863-2378.2008.01145.x 18811908

[B35] Leite-Martins L. Mahú M. Costa A. Mendes A. Lopes E. Mendonça D. (2014). Prevalence of antimicrobial resistance in enteric *Escherichia coli* from domestic pets and assessment of associated risk markers using a generalized linear mixed model. *Prev. Vet. Med.* 117 28–39. 10.1016/j.prevetmed.2014.09.008 25294317

[B36] Leonard E. Pearl D. Janecko N. Finley R. Reid-Smith R. Weese J. (2015). Risk factors for carriage of antimicrobial-resistant *Salmonella* spp and *Escherichia coli* in pet dogs from volunteer households in Ontario, Canada, in 2005 and 2006. *Am. J. Vet. Res.* 76 959–968. 10.2460/ajvr.76.11.959 26512541

[B37] Ludden C. Raven K. Jamrozy D. Gouliouris T. Blane B. Coll F. (2019). One health genomic surveillance of *Escherichia coli* demonstrates distinct lineages and mobile genetic elements in isolates from humans versus livestock. *mBio* 10:e002693–18. 10.1128/mBio.02693-18 30670621 PMC6343043

[B38] Lupo A. Saras E. Madec J. Haenni M. (2018). Emergence of blaCTX-M-55 associated with fosA, rmtB and mcr gene variants in *Escherichia coli* from various animal species in France. *J. Antimicrob. Chemother.* 73 867–872. 10.1093/jac/dkx489 29340602

[B39] Lv L. Partridge S. He L. Zeng Z. He D. Ye J. (2013). ‘Genetic characterization of inci2 plasmids carrying blaCTX-M-55 spreading in both pets and food animals in China Luchao’. *Antimicrob. Agents Chemother.* 57 2824–2827. 10.1128/AAC.02155-12 23478963 PMC3716176

[B40] Magiorakos A. Srinivasan A. Carey R. Carmeli Y. Falagas M. Giske C. (2012). Multidrug-resistant, extensively drug-resistant and pandrug-resistant bacteria: An international expert proposal for interim standard definitions for acquired resistance. *Clin. Microbiol. Infect.* 18 268–281. 10.1111/j.1469-0691.2011.03570.x 21793988

[B41] Matamoros S. van Hattem J. Arcilla M. Willemse N. Melles D. Penders J. (2017). Global phylogenetic analysis of *Escherichia coli* and plasmids carrying the mcr-1 gene indicates bacterial diversity but plasmid restriction. *Sci. Rep.* 7:15364. 10.1038/s41598-017-15539-7 29127343 PMC5681592

[B42] Morgan G. Pinchbeck G. Taymaz E. Chattaway M. Schmidt V. Williams N. (2024). An investigation of the presence and antimicrobial susceptibility of *Enterobacteriaceae* in raw and cooked kibble diets for dogs in the United Kingdom. *Front. Microbiol.* 14:1301841. 10.3389/fmicb.2023.1301841 38260907 PMC10800874

[B43] Morgan G. Williams N. Schmidt V. Cookson D. Symington C. Pinchbeck G. A. (2022). Dog’s Dinner: Factors affecting food choice and feeding practices for UK dog owners feeding raw meat-based or conventional cooked diets. *Prev. Vet. Med.* 208:105741. 10.1016/j.prevetmed.2022.105741 35994979

[B44] Morley P. Strohmeyer R. Tankson J. Hyatt D. Dargatz D. Fedorka-Cray P. (2006). Evaluation of the association between feeding raw meat and *Salmonella enterica* infections at a Greyhound breeding facility. *J. Am. Vet. Med. Assoc.* 228 1524–1532. 10.2460/javma.228.10.1524 16677120

[B45] Mounsey O. Wareham K. Hammond A. Findlay J. Gould V. Morley K. (2022). Evidence that faecal carriage of resistant *Escherichia coli* by 16-week-old dogs in the United Kingdom is associated with raw feeding. *One Health* 14:100370. 10.1016/j.onehlt.2022.100370 35146110 PMC8802057

[B46] Naziri Z. Poormaleknia M. Ghaedi Oliyaei A. (2022). Risk of sharing resistant bacteria and/or resistance elements between dogs and their owners. *BMC Vet. Res.* 18:203. 10.1186/s12917-022-03298-1 35624502 PMC9137046

[B47] Nguyen L. Schmidt H. von Haeseler A. Minh B. Q. (2015). IQ-TREE: A fast and effective stochastic algorithm for estimating maximum-likelihood phylogenies. *Mol. Biol. Evol.* 32 268–274. 10.1093/molbev/msu300 25371430 PMC4271533

[B48] Nilsson O. (2015). Hygiene quality and presence of ESBL-producing *Escherichia coli* in raw food diets for dogs. *Infect. Ecol. Epidemiol.* 5:28758. 10.3402/iee.v5.28758 26490763 PMC4613903

[B49] Nüesch-Inderbinen M. Treier A. Zurfluh K. Stephan R. (2019). Raw meat-based diets for companion animals: A potential source of transmission of pathogenic and antimicrobial-resistant *Enterobacteriaceae*. *R. Soc. Open Sci.* 6:191170. 10.1098/rsos.191170 31824726 PMC6837177

[B50] PDSA (2022). *PDSA animal wellbeing PAW report 2022: The essential insight into the wellbeing of UK pets, PDSA animal wellbeing report.* Birmingham: PDSA.

[B51] Ramos C. Kamei C. Viegas F. de Melo Barbieri J. Cunha J. Hounmanou Y. (2022). Fecal shedding of multidrug resistant *Escherichia coli* isolates in dogs fed with raw meat-based diets in Brazil. *Antibiotics (Basel)* 11:534. 10.3390/antibiotics11040534 35453285 PMC9029118

[B52] Randall L. Clouting C. Horton R. Coldham N. Wu G. Clifton-Hadley F. (2011). Prevalence of *Escherichia coli* carrying extended-spectrum β-lactamases (CTX-M and TEM-52) from broiler chickens and turkeys in Great Britain between 2006 and 2009. *J. Antimicrob. Chemother.* 66 86–95. 10.1093/jac/dkq396 21098542

[B53] Rodríguez-González M. Jiménez-Pearson M. Duarte F. Poklepovich T. Campos J. Araya-Sánchez L. (2020). Multidrug-resistant CTX-M and CMY-2 producing *Escherichia coli* isolated from healthy household dogs from the great Metropolitan Area, Costa Rica. *Microb. Drug Resist.* 26 1421–1428. 10.1089/mdr.2020.0146 33085572 PMC7698996

[B54] Royden A. Ormandy E. Pinchbeck G. Pascoe B. Hitchings M. Sheppard S. (2019). Prevalence of faecal carriage of extended-spectrum β-lactamase (ESBL)-producing *Escherichia coli* in veterinary hospital staff and students. *Vet. Rec. Open* 6:e000307. 10.1136/vetreco-2018-000307 30687506 PMC6327872

[B55] Runesvärd E. Wikström C. Fernström L. Hansson I. (2020). Presence of pathogenic bacteria in faeces from dogs fed raw meat-based diets or dry kibble. *Vet. Rec.* 187:e71. 10.1136/vr.105644 32054718 PMC7799416

[B56] Schmidt V. Pinchbeck G. McIntyre K. Nuttall T. McEwan N. Dawson S. (2018). Routine antibiotic therapy in dogs increases the detection of antimicrobial-resistant faecal *Escherichia coli*. *J. Antimicrob. Chemother.* 73 3305–3316. 10.1093/jac/dky352 30215725

[B57] Schmidt V. Pinchbeck G. Nuttall T. McEwan N. Dawson S. Williams N. (2015). Antimicrobial resistance risk factors and characterisation of faecal *E. coli* isolated from healthy Labrador retrievers in the United Kingdom. *Prev. Vet. Med.* 119 31–40. 10.1016/j.prevetmed.2015.01.013 25732912

[B58] Schmitt K. Kuster S. Zurfluh K. Jud R. Sykes J. Stephan R. (2021). Transmission chains of extended-spectrum beta-lactamase-producing *Enterobacteriaceae* at the companion animal veterinary clinic-household interface. *Antibiotics (Basel)* 10:171. 10.3390/antibiotics10020171 33572066 PMC7914568

[B59] Sealey J. Hammond A. Mounsey O. Gould V. Reyher K. Avison M. (2022). Molecular ecology and risk factors for third-generation cephalosporin-resistant *Escherichia coli* carriage by dogs living in urban and nearby rural settings. *J. Antimicrob. Chemother.* 77 2399–2405. 10.1093/jac/dkac208 35858661 PMC9410662

[B60] Seemann T. (2014). Prokka: Rapid prokaryotic genome annotation. *Bioinformatics* 30 2068–2069. 10.1093/bioinformatics/btu153 24642063

[B61] Segata N. Waldron L. Ballarini A. Narasimhan V. Jousson O. Huttenhower C. (2012). Metagenomic microbial community profiling using unique clade-specific marker genes. *Nat. Methods* 9 811–814. 10.1038/nmeth.2066 22688413 PMC3443552

[B62] Shibu P. McCuaig F. McCartney A. Kujawska M. Hall L. Hoyles L. (2021). Improved molecular characterization of the *Klebsiella oxytoca* complex reveals the prevalence of the kleboxymycin biosynthetic gene cluster. *Microb. Genom.* 7:000592. 10.1099/mgen.0.000592 34142942 PMC8461473

[B63] Sidjabat H. Townsend K. Lorentzen M. Gobius K. Fegan N. Chin J. (2006). Emergence and spread of two distinct clonal groups of multidrug-resistant *Escherichia coli* in a veterinary teaching hospital in Australia. *J. Med. Microbiol.* 55 1125–1134. 10.1099/jmm.0.46598-0 16849734

[B64] Simão F. Waterhouse R. Ioannidis P. Kriventseva E. Zdobnov E. M. (2015). BUSCO: Assessing genome assembly and annotation completeness with single-copy orthologs. *Bioinformatics* 31 3210–3212. 10.1093/bioinformatics/btv351 26059717

[B65] Singleton D. Pongchaikul P. Smith S. Bengtsson R. Baker K. Timofte D. (2021). Temporal, spatial, and genomic analyses of *Enterobacteriaceae* clinical antimicrobial resistance in companion animals reveals phenotypes and genotypes of one health concern. *Front. Microbiol.* 12:700698. 10.3389/fmicb.2021.700698 34394045 PMC8362618

[B66] Sun Y. Zeng Z. Chen S. Ma J. He L. Liu Y. (2010). High prevalence of bla(CTX-M) extended-spectrum β-lactamase genes in *Escherichia coli* isolates from pets and emergence of CTX-M-64 in China. *Clin. Microbiol. Infect.* 16 1475–1481. 10.1111/j.1469-0691.2010.03127.x 21681998

[B67] Tamang M. Nam H. Jang G. Kim S. Chae M. Jung S. (2012). Molecular characterization of extended-spectrum-β-lactamase-producing and plasmid-mediated AmpC β-lactamase-producing *Escherichia coli* isolated from stray dogs in South Korea. *Antimicrob. Agents Chemother.* 56 2705–2712. 10.1128/AAC.05598-11 22354297 PMC3346616

[B68] Timofte D. Maciuca I. Williams N. Wattret A. Schmidt V. (2016). Veterinary hospital dissemination of CTX-M-15 extended-spectrum beta-lactamase-producing *Escherichia coli* ST410 in the United Kingdom. *Microb. Drug Resist.* 22 609–615. 10.1089/mdr.2016.0036 27314838 PMC5073239

[B69] Tonkin-Hill G. MacAlasdair N. Ruis C. Weimann A. Horesh G. Lees J. (2020). Producing polished prokaryotic pangenomes with the Panaroo pipeline. *Genome Biol.* 21:180. 10.1186/s13059-020-02090-4 32698896 PMC7376924

[B70] Toombs-Ruane L. Benschop J. French N. Biggs P. Midwinter A. Marshall J. (2020). Carriage of extended-spectrum-beta-lactamase- and AmpC beta-lactamase-producing *Escherichia coli* strains from humans and pets in the same households. *Appl. Environ. Microbiol.* 86 e1613–e1620. 10.1128/AEM.01613-20 33036993 PMC7688229

[B71] Tuerena I. Williams N. Nuttall T. Pinchbeck G. (2016). Antimicrobial-resistant *Escherichia coli* in hospitalised companion animals and their hospital environment. *J. Small Anim. Pract.* 57 339–347. 10.1111/jsap.12525 27385621

[B72] van den Bunt G. Fluit A. Spaninks M. Timmerman A. Geurts Y. Kant A. (2020). Faecal carriage, risk factors, acquisition and persistence of ESBL-producing *Enterobacteriaceae* in dogs and cats and co-carriage with humans belonging to the same household. *J. Antimicrob. Chemother.* 75 342–350. 10.1093/jac/dkz462 31711228 PMC6966097

[B73] Veterinary Medicines Directorate (2021). *Supplementary material (UK-VARSS 2020). Veterinary antibiotic resistance and sales surveillance report (UK-VARSS 2020).* Addlestone: Veterinary Medicines Directorate.

[B74] Veterinary Medicines Directorate (2022). *UK - veterinary antibiotic resistance and sales surveillance report: 2021. Supplementary material 3 – resistance data’, Veterinary antibiotic resistance and sales surveillance report (UK-VARSS 2021).* Birmingham: Veterinary Medicines Directorate.

[B75] Viegas F. Ramos C. Xavier R. Lopes E. Júnior C. Bagno R. (2020). Fecal shedding of *Salmonella* spp., *Clostridium perfringens*, and *Clostridioides difficile* in dogs fed raw meat-based diets in Brazil and their owners’ motivation. *PLoS One* 15:e0231275. 10.1371/journal.pone.0231275 32287295 PMC7156072

[B76] Wang G. Liu H. Feng Y. Zhang Z. Hu H. Liu J. (2021). Colistin-resistance mcr genes in *Klebsiella pneumoniae* from companion animals. *J. Glob. Antimicrob. Resist.* 25 35–36. 10.1016/j.jgar.2021.02.023 33662641

[B77] Wedley A. Dawson S. Maddox T. Coyne K. Pinchbeck G. Clegg P. (2017). Carriage of antimicrobial resistant *Escherichia coli* in dogs: Prevalence, associated risk factors and molecular characteristics. *Vet. Microbiol.* 199 23–30. 10.1016/j.vetmic.2016.11.017 28110781

[B78] Westgarth C. Pinchbeck G. Bradshaw J. Dawson S. Gaskell R. Christley R. (2008). Dog-human and dog-dog interactions of 260 dog-owning households in a community in Cheshire. *Vet. Rec.* 162 436–442. 10.1136/vr.162.14.436 18390853

[B79] Woodford N. (2008). Successful, multiresistant bacterial clones. *J. Antimicrob. Chemother.* 61 233–234. 10.1093/jac/dkm474 18077310

[B80] Woodford N. Turton J. Livermore D. (2011). Multiresistant Gram-negative bacteria: The role of high-risk clones in the dissemination of antibiotic resistance. *FEMS Microbiol. Rev.* 35 736–755. 10.1111/j.1574-6976.2011.00268.x 21303394

[B81] Zhang J. Zheng B. Zhao L. Wei Z. Ji J. Li L. (2014). Nationwide high prevalence of CTX-M and an increase of CTX-M-55 in *Escherichia coli* isolated from patients with community-onset infections in Chinese county hospitals. *BMC Infect. Dis.* 14:659. 10.1186/s12879-014-0659-0 25466590 PMC4265337

[B82] Zogg A. Simmen S. Zurfluh K. Stephan R. Schmitt S. Nüesch-Inderbinen M. (2018). High prevalence of extended-spectrum β-lactamase producing *Enterobacteriaceae* among clinical isolates from cats and dogs admitted to a veterinary hospital in Switzerland. *Front. Vet. Sci.* 5:62. 10.3389/fvets.2018.00062 29662886 PMC5890143

