# Supplementary material for: Raw meat diets are a major risk factor for carriage of third-generation cephalosporin-resistant and multidrug-resistant E. coli by dogs in the UK
Source: Front Microbiol. 2024 Sep 9;15:1460143. doi: 10.3389/fmicb.2024.1460143 (PMC11417003; doi:10.3389/fmicb.2024.1460143)
Supplement: Supplementary file 1 [file Data_Sheet_1.pdf]

Raw meat diets are an important risk factor for antimicrobial-resistant *E. coli* carriage by dogs in the UK: Appendices

Table A1: *Sequence type, phenotypic antimicrobial resistance as determined by disc diffusion and resistance genes present as determined by whole genome sequencing for extended-spectrum beta-lactamase (ESBL)-producing/third-generation cephalosporin resistant (3GCR)- E. coli isolates (N=75 RMD-fed, N=12 NRMD-fed) from dog faecal samples in the present study NB: No resistance to amikacin, meropenem or tigecycline observed, so omitted from table*

																			**Phenotypic resistance on AST***					
**Sample ID**	**ST**	**Raw YN**	**CTX-M**	**TEM**	**SHV**	**OXA**	***Escherichia coli* ampC1 β-lactamase**	**CMY**	***Escherichia coli* ampC**	***DHA-1***	***qnr***	***parC***	***gyrA***	***tet***	***sul***	***dfrA***	**aminoglycoside resistance genes**	**chloramphenicol resistance genes**	**Amp**	**AmxC**	**Cip**	**TMS**	**Ctx**	**Ctz**
14	10	Yes	32				x		x					B, R			*aph3'')-Ib, aph(6)-Id*		R	S	S	S	R	R
747	10	Yes	15	1			x		x		S1			A	2	14	*aph(3'')-Ib, aph(6)-Id*		R	S	R	R	R	R
533	10	No	1				x		x						2	17	*aadA5*		R	S	S	R	R	S
170	23	Yes		78			x		x					B, R	2				R	R	S	S	S	R
171	23	Yes		78			x		x					B, R	2				R	R	S	R	S	R
655	23	Yes	1				x		x				x		2	17	*aadA5*		R	S	S	R	R	S
600	38	Yes					x	2	x										R	R	S	S	R	R
645	38	Yes	15				x		x		S1			B, R					R	S	S	S	R	R
652	38	Yes	15	52			x		x		S1								R	S	R	S	R	R
653	38	Yes	15				x		x		S1								R	S	R	S	R	R
681	38	Yes	14	1			x		x							1	*aadA2, ant(3'')-IIa*	*catI*	R	S	S	S	R	R
537	48	No	15				x		x		S1				2				R	S	S	S	R	R
193	57	Yes	15	185			x		x					B, R	1				R	S	S	S	R	S
601	58	Yes	15	1			x		x		S1			A	2	14	*aph(3'')-Ib, aph(3')-Ia, aph(6)-Id*		R	S	R	R	R	R

718	58	Yes		52			x	58, 100	x						3	12	*aadA2, aadA25, ant(3'')-IIa*		R	S	S	R	S	S
155	58	No	15	1			x		x		S1			A			*aac(3)-IId, ant(3'')-IIa, aph(3')-Ia*		R	S	R	S	R	R
236	69	Yes	1	1			x		x		S1			B, R			*aac(3)-IId, ant(3'')-IIa , aph(3')-Ia, aph(6)-Id*		R	S	R	S	R	S
207	69	No		1		1, 45	x		x	2	B4			A	1, 2	17	*aadA5, ant(3'')-IIa*		R	R	S	R	S	R
452	75	No					x		x										R	R	S	S	S	R
536	75	No					x		x								*aadA22*		R	R	S	S	R	R
570	88	Yes	14				x		x			x		B, R	1, 2		*ant(3'')-IIa*		R	S	R	R	R	S
173	88	No					x		x						2		*aph(3'')-Ib, aph(6)-Id*		R	R	S	S	S	R
453	88	No		78			x		x				x	B, R	1, 2		*ant(3'')-IIa, aph(3'')-Ib, aph(3')-Ia, aph(6)-Id*		R	R	S	R	S	R
648	101	Yes	55	1			x		x			x	x	A	2	1	*ant(3'')-IIa, aph(3'')-Ib, aph(6)-Id*		R	S	R	R	R	R
649	101	Yes	55	1			x		x			x	x	A	2	1	*ant(3'')-IIa, aph(3'')-Ib, aph(6)-Id*		R	S	R	R	R	R
23	117	Yes		1	66		x		x		S7		x		2, 3	12	*aadA2, aadA25, ant(3'')-IIa*	*cmlA6*	R	S	S	R	S	R
398	117	Yes					x	2	x					A					R	R	S	S	R	R
534	117	Yes		1	66		x		x						2, 3	12	*aadA2, aadA25, ant(3'')-IIa*	*cmlA6*	R	S	S	R	S	R
719	117	Yes		1	66		x		x				x		2, 3	12	*aadA2, aadA5, aadA25, ant(3'')-IIa*		R	S	S	R	S	R
50	117	No					x		x				x						R	R	S	S	R	R
28	155	Yes					x		x										R	R	S	S	S	R

606	155	Yes			66		x	4	x		S1			A			*ant(3'')-IIa*		R	S	S	S	S	R
358	162	Yes	15				x		x			x	x				*ant(3'')-IIa*		R	R	S	R	S	R
385	162	Yes		1	66		x		x			x	x				*ant(3'')-IIa*		R	S	R	S	S	R
654	162	Yes		1			x	2	x			x	x	B, R					R	R	R	S	R	R
31	227	Yes		1			x		x					A	2	14	*aph(3'')-Ib, aph(6)-Id*		R	S	S	R	R	R
715	278	Yes	9				x		x					A	1	16	*aadA2*		R	S	S	R	R	S
482	345	Yes			66		x		x		S1				2				R	S	S	S	S	R
483	345	Yes			66		x		x		S1			A			*aadA17, aph(3'')-Ib*		R	S	S	S	S	R
357	351	Yes	27, 123	135, 185			x		x		S1			A, B, R		1, 14	*aadA8, aadA25, ant(3'')-IIa*		R	S	R	S	R	S
237	362	Yes	2				x	2	x				x	A	1, 2		*ant(3'')-IIa, aph(3')-Ia*	*catI*	R	S	R	R	R	S
175	367	Yes		78			x		x					B, R	2		*aph(3'')-Ib, aph(3')-Ia, aph(6)-Id*		R	R	S	R	S	R
123	372	Yes						2	x										R	R	S	S	R	R
65	399	No					x		x										R	S	R	S	R	R
400	410	Yes					x	2	x										R	R	S	S	R	R
682	442	Yes					x		x								*aadA22, aph(3')-Ia*		R	S	S	S	S	S
138	457	Yes	15				x		x		S1			A			*aac(3)-IId*		R	S	R	S	R	R
607	515	Yes					x	2	x					A	1, 2	12	*aadA2, aph(3'')-Ib, aph(6)-Id*		R	S	S	R	R	R
384	533	Yes			66		x	2	x		S1, S15						*ant(3'')-IIa*		R	S	R	S	S	R
270	540	Yes		1		1	x		x					B, R	3		*aadA2, aadA12, aadA22, aadA23, aadA24, ant(3'')-IIa, aph(3')-Ia*	*catI*	R	R	S	S	R	R
130	602	Yes	1				x		x			x	x	B, R	2	17	*aadA5, aph(3'')-Ib, aph(6)-Id*		R	S	R	R	R	S

189	602	Yes			66		x		x				x	A	2, 3	12	*aadA2, aadA8b, ant(3'')-IIa*	*cmlA6*	R	S	S	R	R	R
272	602	Yes					x	2	x										R	R	S	S	R	R
383	602	Yes					x		x										R	R	S	S	R	R
239	641	Yes	55	1			x	2	x		S1			A	3	14	*aac(3)-IId, ant(3'')-IIa, aph(3')-Ia, aph(6)-Id*	*cmlA6*	R	S	R	R	R	R
176	642	Yes					x		x	2	B4				1	17	*aadA5*		R	R	R	R	S	R
147	752	Yes	55	209			x		x				x	A	2, 3	14	*aadA2, ant(3'')-IIa, aph(3'')-Ib, aph(6)-Id*	*cmlA6*	R	S	R	R	R	R
271	752	Yes	55	150			x		x				x	A	2, 3	1, 14	*aadA3, aadA15, ant(3'')-IIa, aph(3'')-Ib, aph(6)-Id*	*cmlA6*	R	S	R	R	R	R
303	752	Yes	55	209			x		x				x	A	2, 3	14	*aadA2, ant(3'')-IIa, aph(3'')-Ib, aph(6)-Id*	*cmlA6*	R	S	R	R	R	S
361	752	Yes	55, 60	209			x		x				x	A	2, 3	14	*aadA2, ant(3'')-IIa, aph(3'')-Ib, aph(6)-Id*	*cmlA6*	R	S	R	R	R	R
480	752	Yes	55	209			x	6	x				x	A, B(P)	2, 3	14	*aadA2, ant(3'')-IIa, aph(3'')-Ib, aph(6)-Id*	*cmlA6*	R	S	S	R	R	R
685	963	No					x	2, 44	x										R	R	S	S	R	R
194	973	Yes						2	x										R	R	S	S	R	R
603	973	Yes		104, 185				2	x					B, R	2		*aph(3'')-Ib, aph(6)-Id*		R	R	S	R	R	R
359	1081	Yes					x	2	x					B, R	2		*aph(3'')-Ib, aph(6)-Id*		R	R	S	S	S	S
274	1170	Yes	15	1				132						A, M		12	*aadA2, sgm, ant(3'')-IIa*	*cmlA6*	R	S	S	S	R	S

276	1170	Yes	15					132						A, M		12	*aadA2, sgm, ant(3'')-IIa*	*cmlA6*	R	S	S	S	R	S
421	1423	Yes					x		x										R	R	S	S	S	R
360	1508	Yes	32				x		x					B, R			*aac(6')-Ib7, aph(3'')-Ib, aph(6)-Id*		R	S	S	S	R	S
306	1611	Yes	1				x		x										R	S	S	R	R	S
320	1611	Yes	1				x		x										R	S	S	S	R	S
278	1640	Yes	55	1			x		x										R	S	S	S	R	R
643	1727	Yes					x	2	x						2	14	*aph(3'')-Ib, aph(6)-Id*		R	R	S	R	R	R
746	1955	Yes					x	2	x									*cmx*	R	R	S	S	R	R
305	2028	Yes					x		x										R	S	R	S	R	R
304	2171	Yes				45	x	59	x					A			*aph(3')-Ia*		R	R	S	S	R	R
183	2179	Yes	65	1		1	x		x		S2	x	x				*aac(6')-Ib-cr*		R	S	R	S	R	S
567	2705	Yes	24	1			x		x						2	1	*ant(3'')-IIa*		R	S	S	R	R	S
604	2705	Yes					x	2	x										R	R	S	S	R	R
36	4096	Yes	15				x		x		S1			Y			*aph(3')-Ia*		R	R	S	R	S	R
646	4981	No	15	1			x		x			x	x	B, R	2	17	*aadA5, aph(3'')-Ib, aph(3')-Ia, APH(6)-Id*		R	S	R	R	R	R
27	5296	Yes					x		x										R	R	S	S	S	R
136	7483	Yes	15				x		x			x	x	A	1		*aph(3')-Ia*		R	S	R	S	R	R
116	11905	Yes		1	66, 123		x		x		S1			A			*aac(3)-IIe, ant(3'')-IIa, aph(3')-Ia*		R	S	S	S	R	R
538	P1	Yes		60			x										*aac(6')-Iy*		R	S	S	R	R	S
650	P2	Yes	15	1			x		x										R	S	S	S	R	R
651	P2	Yes	15	1			x		x										R	S	S	S	R	R

*Amp: ampicillin; AmxC: amoxycillin-clavulanate; Cip: ciprofloxacin; TMS: trimethoprim-sulphamethoxazole; Ctx: cefotaxime; Ctz: ceftazidime

Table A2: *Univariable analysis of explanatory factors associated with dog faecal carriage of 3GCR-E. coli, analysed at sample level (N=432 dogs). Ref=reference category*

Variable	Category	N (total samples)	% of total	3GCR-*E. coli* present % (N)		Odds ratio	95% CI	p value
				Yes	***No***			
		**432**		**17.4 (75)**	**82.6 (357)**			
1. Food								
Fed raw diet	Yes	193	55.3	32. 6 (63)	67.4 (130)	9.17	4.77, 17.63	<0.001
	No	239	44.7	5.0 (12)	95.0 (227)	*Ref*		
Type of diet fed	Pre-prepared raw yes	157	36.3	34.3 (54)	65.6 (103)	6.34	3.65, 11.03	<0.001
	Pre-prepared raw no	275	63.7	7.6 (21)	92.4 (254)	*Ref*		
	DIY/home-prepared raw yes	101	23.4	29.7 (30)	70.3 (71)	2.69	1.58, 4.56	<0.001
	DIY/home-prepared raw no	331	76.6	13.6 (45)	86.4 (286)	*Ref*		
	Cooked meat/bones yes	43	10.0	9.3 (4)	90.7 (39)	0.46	0.16, 1.33	0.15
	Cooked meat/bones no	389	90.0	18.3 (71)	81.7 (318)	*Ref*		
	Cooked commercial complete dry/kibble yes	254	58.8	7.5 (19)	92.5 (235)	0.18	0.10, 0.31	<0.001
	Cooked commercial complete dry/kibble no	178	41.2	31.5 (56)	68.5 (122)	*Ref*		
	Cooked commercial complete wet yes	108	25.0	4.6 (5)	95.4 (103)	0.18	0.07, 0.45	<0.001
	Cooked commercial complete wet no	324	75.0	21.6 (70)	78.4 (254)	*Ref*		
	Vegetarian/vegan yes	4	0.9	25.0 (1)	75.0 (4)	1.60	0.16, 15.54	0.69
	Vegetarian/vegan no	428	99.1	17.3 (74)	82.7 (354)	*Ref*		
	Other yes	39	9.0	15.4 (6)	84.6 (33)	0.85	0.34, 2.12	0.73
	Other no	393	91.0	17.6 (69)	82.4 (324)	*Ref*		
Diet changed in last 3 months	Yes	83	19.2	15.7 (13)	84.3 (70)	0.87	0.45, 1.67	0.68
	No	347	80.3	17.6 (61)	82.4 (286)	*Ref*		
	Unknown	2	0.5	50.0 (1)	50.0 (1)	NA	NA	
Types of treat fed	Shop bought cooked treats/biscuits yes	273	63.2	11.4 (31)	88.6 (242)	0.33	0.20, 0.56	<0.001

	Shop bought cooked treats/biscuits no	159	36.8	27.2 (44)	72.3 (115)	*Ref*		
	Freeze dried treats yes	136	31.5	27.9 (38)	72.1 (98)	2.71	1.63, 4.52	<0.001
	Freeze dried treats no	296	68.5	12.5 (37)	87.5 (259)	*Ref*		
	Dried treats yes	159	36.8	24.5 (39)	75.5 (120)	2.14	1.29, 3.54	<0.01
	Dried treats no	273	63.2	13.2 (36)	86.8 (237)	*Ref*		
	Raw meat yes	75	17.4	32.0 (24)	68.0 (51)	2.82	1.60, 4.99	<0.001
	Raw meat no	357	82.6	14.3 (51)	85.7 (306)	*Ref*		
	Raw bones yes	97	22.5	30.9 (30)	69.1 (67)	2.89	1.69, 4.92	<0.001
	Raw bones no	335	77.5	13.4 (45)	86.6 (290)	*Ref*		
	Cooked meat yes	104	24.1	12.5 (13)	87.5 (91)	0.61	0.32, 1.17	0.14
	Cooked meat no	328	75.9	18.9 (62)	81.1 (266)	*Ref*		
	Cooked bones yes	10	2.3	10.0 (1)	90.0 (9)	0.52	0.07, 4.19	0.54
	Cooked bones no	422	97.7	17.5 (74)	82.5 (348)	*Ref*		
	I don't feed any treats yes	16	3.7	12.5 (2)	87.5 (14)	0.67	0.15, 3.01	0.60
	I don't feed any treats no	416	96.3	17.5 (73)	82.5 (343)	*Ref*		
	Other treats yes	110	25.5	16.4 (18)	83.6 (92)	0.91	0.51, 1.63	0.75
	Other treats no	322	74.5	17.7 (57)	82.3 (265)	*Ref*		
Human food/titbits given	Frequently	70	16.2	11.4 (8)	88.6 (62)	0.57	0.23, 1.43	0.13
	Occasionally as a treat	169	39.1	14.8 (25)	85.2 (144)	0.76	0.38, 1.54	
	Rarely	109	25.2	23.9 (26)	76.1 (83)	1.38	0.68, 2.81	
	No	81	18.8	18.5 (15)	81.5 (66)	*Ref*		
	Unknown	3	0.7	33.3 (1)	66.7 (2)	NA		
Dog scavenges	Yes frequently	74	17.1	13.5 (10)	86.5 (64)	0.71	0.33, 1.53	0.64
	Yes sometimes	165	38.2	18.2 (30)	81.8 (135)	1.01	0.59, 1.74	
	No	189	43.8	18.0 (34)	82.0 (155)	*Ref*		
	Unknown	4	0.9	25.0 (1)	75.0 (3)	NA		
2. Antibiotic use								
Antibiotics in last 3 months	Yes	47	10.9	27.7 (13)	72.3 (34)	1.99	1.00, 3.99	0.05
	No	385	89.1	16.1 (62)	83.9 (323)	*Ref*		
Currently receiving antibiotics	Yes	5	1.2	20.0 (1)	80.0 (4)	1.21	0.13, 10.97	0.87

	No	426	98.6	17.1 (73)	82.9 (353	*Ref*		
	Unknown	1	0.2	100.0 (1)	0.0 (0)	NA		
Most recent antibiotic course	1 week ago or less	4	0.9	50.0 (2)	50.0 (2)	5.23	0.73, 38.03	0.18
	2-8 weeks ago	17	3.9	23.5 (4)	76.5 (13)	1.62	0.51, 5.13	
	More than 8 weeks ago	22	5.1	27.3 (6)	72.7 (16)	1.97	0.74, 5.23	
	Not applicable	388	89.8	16.0 (62)	84.0 (326)	*Ref*		
	Unknown	1	0.2	100.0 (1)	0.0 (0)	NA		
Duration of most recent course	One off injection	3	0.7	33.3 (1)	66.7 (2)	2.65	0.24, 29.66	0.09
	Oral antibiotics up to 5 days	16	3.7	18.8 (3)	81.3 (13)	1.22	0.34, 4.42	
	Oral antibiotics up to 10 days	20	4.6	40.0 (8)	60.0 (12)	3.53	1.39, 8.90	
	Oral antibiotics for 2 weeks or longer	7	1.6	28.6 (2)	71.4 (5)	2.12	0.40, 11.17	
	Not applicable	384	88.9	15.9 (61)	84.1 (323)	*Ref*		
	Unknown	2	0.5	0.0 (0)	100.0 (2)	NA		
3. Diarrhoea								
Diarrhoea/loose stools last 3 months	Yes	138	31.9	14.5 (20)	85.5 (118)	0.73	0.42, 1.28	0.28
	No	293	67.8	18.8 (55)	81.2 (238)	*Ref*		
	Unknown	1	0.2	0.0 (0)	100.0 (1)			
Most recent episode	Currently has/always has/in the last week	18	4.2	5.6 (1)	94.4 (17)	0.26	0.03, 1.99	0.73
	1-2 weeks ago	35	8.1	20.0 (7)	80.0 (28)	1.10	0.46, 2.66	
	2-4 weeks ago	36	8.3	16.7 (6)	83.3 (30)	0.88	0.35, 2.22	
	4-8 weeks ago	28	6.5	10.7 (3)	89.3 (25)	0.53	0.15, 1.82	
	More than 8 weeks ago	21	4.9	19.0 (4)	81.0 (17)	1.04	0.34, 3.21	
	Not applicable	292	67.6	18.5 (54)	81.5 (238)	*Ref*		
	Unknown	2	0.5	0.0 (0)	100.0 (2)	NA		
Repeated episodes in last 3 months	Yes	46	10.6	10.9 (5)	89.1 (41)	0.53	0.20, 1.40	0.42
	No	91	21.1	16.5 (15)	82.5 (76)	0.85	0.46, 1.60	
	Not applicable	293	67.8	18.8 (55)	81.2 (238)	*Ref*		
	Unknown	2	0.5	0.0 (0)	100.0 (2)	NA		
Number of episodes in last 3 months	Constant diarrhoea/up to 2 episodes	26	6.0	7.7 (2)	92.3 (24)	0.38	0.09, 1.64	0.41

	3-4 episodes	15	3.5	26.7 (4)	73.3 (11)	1.66	0.51, 5.36	
	5 or more episodes	17	3.9	11.8 (2)	88.2 (15)	0.61	0.14, 2.72	
	Not applicable	372	86.1	18.0 (67)	82.0 (305)	*Ref*		
	Unknown	2	0.5	0.0 (0)	100.0 (2)	NA		
Treatment given	None, resolved by itself (yes)	71	16.4	14.1 (10)	85.9 (61)	0.75	0.36, 1.53	0.43
	None, resolved by itself (no)	361	83.6	18.0 (65)	82.0 (296)	*Ref*		
	Bland diet (yes)	25	5.8	16.0 (4)	84.0 (21)	0.90	0.30, 2.71	0.85
	Bland diet (no)	407	94.2	17.4 (71)	82.6 (336)	*Ref*		
	Home remedy (yes)	11	2.5	27.5 (3)	72.7 (8)	1.82	0.47, 7.02	0.39
	Home remedy (no)	421	97.5	17.1 (72)	82.9 (349)	*Ref*		
	Over the counter medication from a shop (yes)	25	5.8	4.0 (1)	96.0 (24)	0.19	0.03, 1.41	0.10
	Over the counter medication from a shop (no)	407	94.2	18.2 (74)	81.8 (333)	*Ref*		
	Veterinary prescribed treatment (yes)	19	4.4	10.5 (2)	89.5 (17)	0.55	0.12, 2.42	0.43
	Veterinary prescribed treatment (no)	413	95.6	17.7 (73)	82.3 (340)	*Ref*		
4.Vet visits								
Visit to vet in the last 3 months	Yes	189	43.8	17.5 (33)	82.5 (156)	1.01	0.61, 1.67	0.98
	No	242	46.0	17.4 (42)	82.6 (200)	*Ref*		
	Unknown	1	0.2	0.0 (0)	100.0 (0)	NA		
Number of vet visits	1	102	23.6	16.7 (17)	83.3 (85)	0.98	0.53, 1.81	0.83
	2	43	10.0	14.0 (6)	86.0 (37)	0.79	0.31, 2.00	
	3	22	5.1	22.7 (5)	77.3 (17)	1.44	0.50, 4.11	
	4	8	1.9	12.5 (1)	87.5 (7)	0.70	0.08, 5.82	
	5 or more visits	14	3.2	28.6 (4)	71.4 (10)	1.95	0.58, 6.53	
	Not applicable	241	55.8	17.0 (41)	83.0 (200)	*Ref*		
	Unknown	2	0.5	50.0 (1)	50.0 (1)	NA		
Reason for visit	Emergency	16	3.7	43.8 (7)	56.3 (9)	3.79	1.34, 10.77	0.06
	Non-emergency problem/concern	111	25.7	14.4 (16)	85.6 (95)	0.82	0.44, 1.54	
	Routine visit	62	14.4	17.7 (11)	82.3 (51)	1.05	0.51, 2.19	
	Not applicable	241	55.8	17.0 (41)	83.0 (17)	*Ref*		
	Unknown	2	0.5	0.0 (0)	100.0 (2)	NA		

Patient hospitalised	For the day only	32	7.4	12.5 (4)	87.5 (28)	0.70	0.23, 2.10	0.73
	For longer than 24 hours	3	0.7	33.3 (1)	66.7 (2)	2.45	0.22, 27.67	
	No	153	35.4	19.0 (29)	81.0 (124)	1.15	0.69, 1.94	
	Not applicable	242	56.0	16.9 (41)	83.1 (124)	*Ref*		
	Unknown	2	0.5	0.0 (0)	100.0 (2)	NA		
5. Preventative healthcare and exposure to other animals and carehomes								
Antiparasite treatment given	No treatment (yes)	69	16.0	31.9 (22)	68.1 (47)	2.74	1.52, 4.91	<0.001
	No treatment (no)	363	84.0	14.6 (53)	85.4 (310)	*Ref*		
	Vet prescribed treatment (yes)	264	61.1	11.7 (31)	88.3 (233)	0.38	0.23, 0.62	<0.001
	Vet prescribed treatment (no)	168	38.9	26.2 (44)	73.8 (124)	*Ref*		
	Over the counter/shop bought treatment (yes)	41	9.5	12.2 (5)	87.8 (36)	0.64	0.24, 1.68	0.36
	Over the counter/shop bought treatment (no)	391	90.5	17.9 (70)	82.1 (321)	*Ref*		
	Natural remedy (yes)	72	16.7	25.0 (18)	75.0 (54)	1.77	0.97, 3.24	0.06
	Natural remedy (no)	360	83.3	15.8 (57)	84.2 (303)	*Ref*		
Regular contact with other animals	Dogs (yes)	380	88.0	17.1 (65)	82.9 (315)	0.88	0.41, 1.82	0.70
	Dogs (no)	52	12.0	19.2 (10)	80.8 (42)	*Ref*		
	Cats (yes)	149	34.5	12.1 (18)	87.9 (131)	0.55	0.31, 0.97	0.04
	Cats (no)	283	65.5	20.1 (57)	79.9 (226)	*Ref*		
	Small mammals/rodents (yes)	56	13.0	12.5 (7)	87.5 (49)	0.65	0.28, 1.49	0.31
	Small mammals/rodents (no)	376	87.0	18.1 (68)	81.9 (308)	*Ref*		
	Horses (yes)	81	18.8	13.6 (11)	86.4 (70)	0.71	0.35, 1.41	0.32
	Horses (no)	351	81.3	18.2 (64)	81.8 (287)	*Ref*		
	Farm animals (yes)	75	17.4	13.3 (10)	86.7 (65)	0.69	0.34, 1.42	0.31
	Farm animals (no)	357	82.6	18.2 (65)	81.8 (292)	*Ref*		
	Wildlife (yes)	126	29.2	16.7 (21)	83.3 (105)	0.93	0.54, 1.62	0.81
	Wildlife (no)	306	70.8	17.6 (54)	82.4 (252)	*Ref*		
	Reptiles/snakes (yes)	13	3.0	15.4 (2)	84.6 (11)	0.86	0.19, 3.97	0.85
	Reptiles/snakes (no)	419	97.0	17.4 (73)	82.6 (346)	*Ref*		
	Chickens/poultry (yes)	29	6.7	13.8 (4)	86.2 (25)	0.75	0.25, 2.22	0.60
	Chickens/poultry (no)	403	93.3	17.6 (71)	82.4 (332)	*Ref*		

	No other regular contact (yes)	26	6.0	23.1 (6)	76.9 (20)	1.47	0.57, 3.78	0.43
	No other regular contact (no)	406	94.0	17.0 (69)	83.0 (337)	*Ref*		
	Other (yes)	41	9.5	14.6 (6)	85.4 (35)	0.80	0.32, 1.98	0.63
	Other (no)	391	90.5	17.6 (69)	82.4 (332)	*Ref*		
Regular access to communal areas	Dog training classes (yes)	77	17.8	23.4 (18)	76.6 (59)	1.60	0.88, 2.90	0.13
	Dog training classes (no)	355	82.2	16.1 (57)	83.9 (298)	*Ref*		
	Doggy daycare (yes)	26	6.0	30.8 (8)	69.2 (18)	2.25	0.94, 5.38	0.07
	Doggy daycare (no)	406	94.0	16.5 (67)	83.5 (339)	*Ref*		
	Group dog walking (yes)	67	15.5	22.4 (15)	77.6 (52)	1.47	0.78, 2.77	0.24
	Group dog walking (no)	365	84.5	16.4 (60)	83.6 (305)	*Ref*		
	Dog shows (yes)	40	9.3	32.5 (13)	67.5 (27)	2.56	1.25, 5.22	0.01
	Dog shows (no)	391	90.7	15.9 (62)	84.1 (329)	*Ref*		
	Dog parks (yes)	77	17.8	14.3 (11)	85.7 (66)	0.76	0.38, 1.52	0.43
	Dog parks (no)	355	82.2	18.0 (64)	82.0 (291)	*Ref*		
	Farm land (yes)	222	51.4	15.8 (35)	84.2 (187)	0.80	0.48, 1.31	0.37
	Farm land (no)	210	48.6	19.0 (40)	81.0 (170)	*Ref*		
	Public parks/towpaths/footpaths (yes)	354	81.9	17.8 (63)	82.2 (291)	1.19	0.61, 2.33	0.61
	Public parks/towpaths/footpaths (no)	78	18.1	15.4 (12)	84.6 (66)	*Ref*		
	Other (yes)	70	16.2	17.1 (12)	82.9 (58)	0.98	0.50, 1.94	0.96
	Other (no)	362	83.8	17.4 (63)	82.6 (299)	*Ref*		
	No regular access to places listed (yes)	18	4.2	5.6 (1)	94.4 (17)	0.27	0.04, 2.06	0.21
	No regular access to places listed (no)	414	95.8	17.9 (74)	82.1 (340)	*Ref*		
Visit human carehomes (e.g. PAT dog)	Yes	8	1.9	37.5 (3)	62.5 (5)	2.91	0.68, 12.4	0.15
	No	421	97.5	17.1 (72)	82.9 (349)	*Ref*		
	Unknown	3	0.7	0.0 (0)	100.0 (3)	NA		
6. Household data								
Number of people in household	1	66	15.3	19.7 (13)	80.3 (53)	*Ref*		0.92
	2	220	50.9	18.2 (40)	81.8 (180)	0.91	0.45, 1.82	
	3	69	16.0	15.9 (11)	84.1 (58)	0.77	0.32, 1.87	
	4	58	13.4	13.8 (8)	86.2 (50)	0.65	0.25, 1.71	
	5 or more	17	3.9	17.6 (3)	82.4 (14)	0.87	0.22, 3.50	

	Unknown	2	0.5	0.0 (0)	100.0 (2)	NA		
Residents present aged 65 or over?	Yes	87	20.1	18.4 (16)	81.6 (71)	1.08	0.59, 1.99	0.80
	No	382	79.2	17.3 (59)	82.7 (283)	*Ref*		
	Unknown	3	0.7	0.0 (0)	100.0 (3)	NA		
Residents present aged 5 or younger?	Yes	27	6.3	3.7 (1)	96.3 (26)	0.17	0.02, 1.28	0.09
	No	402	93.1	18.4 (74)	81.6 (328)	*Ref*		
	Unknown	3	0.7	0.0 (0)	100.0 (3)	NA		
Resident works in riskier areas	Hospital/GP surgery (yes)	27	6.3	25.9 (7)	74.1 (20)	1.74	0.71, 4.26	0.23
	Hospital/GP surgery (no)	405	93.8	16.8 (68)	83.2 (337)	*Ref*		
	Carehome (yes)	8	1.9	25.0 (2)	75.0 (6)	1.6	0.32, 8.10	0.57
	Carehome (no)	424	98.1	17.2 (73)	82.3 (351)	*Ref*		
	Nursery (yes)	3	0.7	66.7 (2)	33.3 (1)	9.75	0.87, 108.99	0.06
	Nursery (no)	429	99.3	17.0 (73)	83.0 (356)	*Ref*		
	Primary school (yes)	11	2.5	27.3 (3)	72.7 (8)	1.82	0.47, 7.02	0.37
	Primary school (no)	421	97.5	17.1 (72)	82.9 (349)	*Ref*		
-	Livestock farm (yes)	13	3.0	15.4 (2)	84.6 (11)	0.86	0.19, 3.97	0.85
	Livestock farm (no)	419	97.0	17.4 (73)	82.6 (346)	*Ref*		
	Dog boarding kennels (yes)	9	2.1	33.3 (3)	66.7 (6)	2.44	0.60, 9.97	0.22
	Dog boarding kennels (no)	423	97.9	17.0 (72)	83.0 (351)	*Ref*		
	Petting zoo (yes)	2	0.5	50.0 (1)	50.0 (1)	4.81	0.30, 77.79	0.27
	Petting zoo (no)	430	99.5	17.2 (74)	82.8 (356)	*Ref*		
	Veterinary practice (yes)	113	26.2	8.0 (9)	92.0 (104)	0.33	0.16, 0.69	0.00
	Veterinary practice (no)	319	73.8	20.7 (66)	79.3 (253)	*Ref*		
	No other risky workplace (yes)	259	60.0	19.3 (50)	80.7 (209)	1.42	0.84, 2.39	0.19
	No other risky workplace (no)	173	40.0	14.5 (25)	85.5 (148)	*Ref*		
Resident received antibiotics last 3 months	Yes	51	11.8	15.7 (8)	84.3 (43)	0.86	0.39, 1.92	0.71
	No	377	87.3	17.8 (67)	82.2 (310)	*Ref*		
	Unknown	4	0.9	0.0 (0)	100.0 (4)	NA		
Resident hospitalised in last 3 months	Yes	15	3.5	13.3 (2)	86.7 (13)	0.71	0.16, 3.23	0.66

	No	412	95.4	17.7 (73)	82.3 (339)	*Ref*		
	Unknown	5	1.2	0.0 (0)	100.0 (5)	NA		
Region of country	East Midlands	28	6.5	14.4 (4)	85.7 (24)	2.06	0.42, 10.01	0.37
	East of England	27	6.3	14.8 (4)	85.2 (23)	2.15	0.44, 10.46	
	Greater London	12	2.8	33.3 (4)	66.7 (8)	6.17	1.15, 33.11	
	North East and Yorkshire	40	9.3	5.0 (2)	95.0 (38)	*Ref*		
	North West	104	24.1	14.4 (15)	85.6 (89)	2.08	0.59, 7.61	
	Northern Ireland	4	0.9	50.0 (2)	50.0 (2)	12.33	1.26, 121.30	
	Scotland	20	4.6	15.0 (3)	85.0 (17)	2.18	0.40, 11.92	
	South East	73	16.9	20.5 (15)	79.5 (58)	3.19	0.86, 11.78	
	South West	47	10.9	25.5 (12)	74.5 (35)	4.23	1.10, 16.26	
	Wales	30	6.9	20.0 (6)	80.0 (24)	3.08	0.70, 13.52	
	West Midlands	36	8.3	13.9 (5)	86.1 (31)	1.99	0.44, 8.99	
	Unknown	11	2.5	18.2 (2)	81.8 (9)	NA		
7. Dog data								
Dog sex	Female entire	48	11.1	29.2 (14)	70.8 (34)	2.78	1.29, 6.03	0.08
	Female neutered	163	37.7	12.9 (21)	87.1 (142)	*Ref*		
	Male entire	68	15.7	19.1 (13)	80.9 (55)	1.60	0.75, 3.41	
	Male neutered	150	34.7	18.0 (27)	82.0 (123)	1.48	0.80, 2.76	
	Unknown	3	0.7	0.0 (0)	100.0 (3)	NA		
Dog age	<12 months	29	6.7	24.1 (7)	75.9 (22)	0.92	0.86, 0.99	0.02
	1 year	16	3.7	6.3 (1)	93.8 (15)			
	2 years	42	9.7	28.6 (12)	71.4 (30)			
	3 years	38	8.8	31.6 (12)	68.4 (26)			
	4 years	36	8.3	8.3 (3)	91.7 (33)			
	5 years	37	8.6	18.9 (7)	81.1 (30)			

	6 years	36	8.3	25.0 (9)	75.0 (27)			
	7 years	34	7.9	14.7 (5)	85.3 (29)			
	8 years or older	157	36.9	12.1 (19)	87.9 (138)			
	Unknown	7	1.6	0.0 (0)	100.0 (7)	NA		
Dog age[2]	<12 months	29	6.7	24.1 (7)	75.9 (22)	0.99	0.99, 1.00	0.24
	1 year	16	3.7	6.3 (1)	93.8 (15)			
	2 years	42	9.7	28.6 (12)	71.4 (30)			
	3 years	38	8.8	31.6 (12)	68.4 (26)			
	4 years	36	8.3	8.3 (3)	91.7 (33)			
	5 years	37	8.6	18.9 (7)	81.1 (30)			
	6 years	36	8.3	25.0 (9)	75.0 (27)			
	7 years	34	7.9	14.7 (5)	85.3 (29)			
	8 years or older	157	36.9	12.1 (19)	87.9 (138)			
	Unknown	7	1.6	0.0 (0)	100.0 (7)	NA		
Length of time owned (8+ combined)	<12 months	44	10.4	22.7 (10)	77.3 (34)	0.91	0.84, 0.98	0.02
	1 year	23	5.4	13.0 (3)	87.0 (20)			
	2 years	49	11.5	24.5 (12)	75.5 (37)			
	3 years	43	10.1	27.9 (12)	72.1 (31)			
	4 years	57	13.4	19.3 (11)	80.7 (46)			
	5 years	36	8.5	22.2 (8)	77.8 (28)			
	6 years	31	7.3	12.9 (4)	87.1 (27)			
	7 years	28	6.6	10.7 (3)	89.3 (25)			
	8 years or longer	114	26.8	10.5 (12)	89.5 (102)			
	Unknown	7	1.6	0.0 (0)	100.0 (7)	NA		

Table A3: *Univariable analysis of explanatory factors associated with dog faecal carriage of ESBL-producing E. coli, analysed at sample level (N=432 dogs). Ref=reference category*

**Variable**	**Category**	**N (total samples)**	**% of total**	**ESBL-*E. coli* present % (N)**		**Odds ratio**	**95% CI**	**p value**
				Yes	***No***			
		**432**		**11.8 (51)**	**88.2 (381)**			
1. Food								
Fed raw diet	Yes	193	55.3	24.4 (47)	75.6 (146)	18.91	6.68, 53.59	<0.001
	No	239	44.7	1.7 (4)	98.3 (235)			
Type of diet fed	Pre-prepared raw yes	157	36.3	24.8 (39)	75.2 (118)	7.24	3.66, 14.33	<0.001
	Pre-prepared raw no	275	63.7	4.4 (12)	95.6 (263)	*Ref*		
	DIY/home-prepared raw yes	101	23.4	21.8 (22)	78.2 (79)	2.90	1.58, 5.32	<0.001
	DIY/home-prepared raw no	331	76.6	8.8 (29)	91.2 (302)	*Ref*		
	Cooked meat/bones yes	43	10.0	4.7 (2)	95.3 (41)	0.34	0.08, 1.44	0.14
	Cooked meat/bones no	389	90.0	12.6 (49)	87.4 (340)	*Ref*		
	Cooked commercial complete dry/kibble yes	254	58.8	3.1 (8)	96.9 (246)	0.10	0.05, 0.22	<0.001
	Cooked commercial complete dry/kibble no	178	41.2	24.2 (43)	75.8 (135)	*Ref*		
	Cooked commercial complete wet yes	108	25.0	2.8 (3)	97.2 (105)	0.16	0.05, 0.54	0.00
	Cooked commercial complete wet no	324	75.0	14.8 (48)	85.2 (276)	*Ref*		
	Vegetarian/vegan yes	4	0.9	25.0 (1)	75.0 (3)	2.52	0.26, 24.69	0.43
	Vegetarian/vegan no	428	99.1	11.7 (50)	88.3 (378)	*Ref*		
	Other yes	39	9.0	5.1 (2)	94.9 (37)	0.38	0.09, 1.62	0.19
	Other no	393	91.0	12.5 (49)	87.5 (344)	*Ref*		
Diet changed in last 3 months	Yes	83	19.2	4.8 (4)	95.2 (79)	0.33	0.12, 0.95	0.04
	No	347	80.3	13.3 (46)	86.7 (301)	*Ref*		
	Unknown	2	0.5	50.0 (1)	50.0 (1)	NA		
Types of treat fed	Shop bought cooked treats/biscuits yes	273	63.2	6.6 (18)	83.4 (255)	0.27	0.15, 0.50	<0.001
	Shop bought cooked treats/biscuits no	159	36.8	20.8 (33)	79.2 (126)	*Ref*		
	Freeze dried treats yes	136	31.5	19.9 (27)	80.1 (109)	2.81	1.55, 5.08	<0.001

	Freeze dried treats no	296	68.5	8.1 (24)	91.9 (272)	*Ref*			
	Dried treats yes	159	36.8	15.7 (25)	84.3 (134)		1.77	0.99, 3.19	0.06
	Dried treats no	273	63.2	9.5 (26)	90.5 (247)	*Ref*			
	Raw meat yes	75	17.4	18.7 (14)	81.3 (61)		1.99	1.01, 3.89	0.05
	Raw meat no	357	82.6	10.4 (37)	89.6 (320)	*Ref*			
	Raw bones yes	97	22.5	19.6 (19)	80.4 (78)		2.31	1.24, 4.29	0.01
	Raw bones no	335	77.5	9.6 (32)	90.4 (303)	*Ref*			
	Cooked meat yes	104	24.1	7.7 (8)	92.3 (96)		0.55	0.25, 1.22	0.14
	Cooked meat no	328	75.9	13.1 (43)	86.9 (285)	*Ref*			
	Cooked bones yes	10	2.3	0.0 (0)	100.0 (10)				0.62
	Cooked bones no	422	97.7	12.1 (51)	87.9 (371)	*Ref*			
	I don't feed any treats yes	16	3.7	12.5 (2)	87.5 (14)		1.07	0.24, 4.85	0.93
	I don't feed any treats no	416	96.3	11.8 (49)	88.2 (367)	*Ref*			
	Other treats yes	110	25.5	13.6 (15)	86.4 (95)		1.25	0.66, 2.39	0.49
	Other treats no	322	74.5	11.2 (36)	88.8 (286)	*Ref*			
Human food/titbits given	Frequently	70	16.2	5.7 (4)	94.3 (66)		0.55	0.16, 1.92	0.01
	Occasionally as a treat	169	39.1	8.9 (15)	91.1 (154)		0.89	0.36, 2.19	
	Rarely	109	25.2	21.1 (23)	78.9 (86)		2.44	1.03, 5.78	
	No	81	18.8	9.9 (8)	90.1 (73)	*Ref*			
	Unknown	3	0.7	33.3 (1)	66.7 (2)	NA			
Dog scavenges	Yes frequently	74	17.1	8.1 (6)	91.9 (68)		0.67	0.26, 1.72	0.51
	Yes sometimes	165	38.2	13.3 (22)	86.7 (143)		1.17	0.62, 2.20	
	No	189	43.8	11.6 (22)	88.4 (167)	*Ref*			
	Unknown	4	0.9	25.0 (1)	75.0 (3)	NA			
2. Antibiotic use									
Antibiotics in last 3 months	Yes	47	10.9	21.3 (10)	78.7 (37)		2.27	1.05, 4.90	0.04
	No	385	89.1	10.6 (41)	89.4 (344)	*Ref*			
Currently receiving antibiotics	Yes	5	1.2	20.0 (1)	80.0 (4)		1.92	0.21, 17.56	0.56
	No	426	98.6	11.5 (49)	88.5 (377)	*Ref*			
	Unknown	1	0.2	100.0 (1)	0.0 (0)	NA			

Most recent antibiotic course	Less than 8 weeks ago	21	4.9	19.0 (4)	81.0 (17)	1.94	0.62, 6.03	0.32
	More than 8 weeks ago	22	5.1	18.2 (4)	81.8 (18)	1.83	0.59, 5.67	
	Not applicable	388	89.8	10.8 (42)	89.2 (346)	*Ref*		
	Unknown	1	0.2	100.0 (1)	0.0 (0)	NA		
Duration of most recent course	One off injection	3	0.7	33.3 (1)	66.7 (2)	4.18	0.37, 47.14	0.17
	Oral antibiotics up to 5 days	16	3.7	12.5 (2)	87.5 (14)	1.20	0.26, 5.45	
	Oral antibiotics up to 10 days	20	4.6	25.0 (5)	75.0 (15)	2.79	0.96, 8.07	
	Oral antibiotics for 2 weeks or longer	7	1.6	28.6 (2)	71.4 (5)	3.35	0.63, 17.80	
	Not applicable	384	88.9	10.7 (41)	89.3 (343)	*Ref*		
	Unknown	2	0.5			NA		
3. Diarrhoea								
Diarrhoea/loose stools last 3 months	Yes	138	31.9	9.4 (13)	90.6 (125)	0.70	0.36, 1.36	0.24
	No	293	67.8	13.0 (38)	87.0 (255)	*Ref*		
	Unknown	1	0.2	0.0 (0)	100.0 (1)	NA		
Most recent episode	Currently has/always has/in the last week	18	4.2	5.6 (1)	94.4 (17)	0.41	0.05, 3.14	0.91
	1-2 weeks ago	35	8.1	11.4 (4)	88.6 (31)	0.89	0.30, 2.66	
	2-4 weeks ago	36	8.3	11.1 (4)	88.9 (32)	0.86	0.29, 2.58	
	4-8 weeks ago	28	6.5	7.1 (2)	92.9 (26)	0.53	0.12, 2.33	
	More than 8 weeks ago	21	4.9	14.3 (3)	85.7 (18)	1.15	0.32, 4.09	
	Not applicable	292	67.6	12.7 (37)	87.3 (255)	*Ref*		
	Unknown	2	0.5	0.0 (0)	100.0 (2)	NA		
Repeated episodes in last 3 months	Yes	46	10.6	4.3 (2)	95.7 (44)	0.31	0.07, 1.31	0.28
	No	91	21.1	12.1 (11)	87.9 (80)	0.92	0.45, 1.89	
	Not applicable	293	67.8	13.0 (38)	87.0 (255)	*Ref*		
	Unknown	2	0.5	0.0 (0)	100.0 (2)	NA		
Number of episodes in last 3 months	Constant diarrhoea/up to 2 episodes	26	6.0	3.8 (1)	96.2 (25)	0.28	0.04, 2.09	0.54
	3-4 episodes	15	3.5	13.3 (2)	86.7 (13)	1.06	0.23, 4.86	
	5 or more episodes	17	3.9	5.9 (1)	94.1 (16)	0.43	0.06, 3.34	
	Not applicable	372	86.1	12.6 (47)	87.4 (325)	*Ref*		
	Unknown	2	0.5	0.0 (0)	100.0 (2)	NA		

Treatment given	None, resolved by itself (yes)	71	16.4	9.9 (7)	90.1 (64)	0.79	0.34, 1.83	0.58
	None, resolved by itself (no)	361	83.6	12.2 (44)	87.8 (317)	*Ref*		
	Bland diet (yes)	25	5.8	8.0 (2)	92.0 (23)	0.64	0.15, 2.78	0.55
	Bland diet (no)	407	94.2	12.0 (49)	88.0 (358)	*Ref*		
	Home remedy (yes)	11	2.5	18.2 (2)	81.8 (9)	1.69	0.35, 8.04	0.51
	Home remedy (no)	421	97.5	11.6 (49)	88.4 (372)	*Ref*		
	Over the counter medication from a shop (yes)	25	5.8	0.0 (0)	100.0 (25)			0.06
	Over the counter medication from a shop (no)	407	94.2	12.5 (51)	87.5 (356)	*Ref*		
	Veterinary prescribed treatment (yes)	19	4.4	10.5 (2)	89.5 (17)	0.87	0.20, 3.90	0.86
	Veterinary prescribed treatment (no)	413	95.6	11.9 (49)	88.1 (364)	*Ref*		
4.Vet visits								
Visit to vet in the last 3 months	Yes	189	43.8	13.2 (25)	86.8 (164)	1.27	0.71, 2.27	0.43
	No	242	46.0	10.7 (26)	89.3 (216)	*Ref*		
	Unknown	1	0.2	0.0 (0)	100.0 (1)	NA		
Number of vet visits	1	102	23.6	14.7 (15)	85.3 (87)	1.49	0.75, 2.96	0.91
	2	43	10.0	11.6 (5)	88.4 (38)	1.14	0.41, 3.15	
	3	22	5.1	9.1 (2)	90.9 (20)	0.86	0.91, 3.92	
	4	8	1.9	12.5 (1)	87.5 (7)	1.23	0.15, 10.45	
	5 or more visits	14	3.2	14.3 (2)	85.7 (12)	1.44	0.31, 6.81	
	Not applicable	241	55.8	10.4 (25)	89.6 (216)	*Ref*		
	Unknown	2	0.5	50.0 (1)	50.0 (1)	NA		
Reason for visit	Emergency	16	3.7	37.5 (6)	62.5 (10)	5.18	1.74, 15.47	0.03
	Non-emergency problem/concern	111	25.7	11.7 (13)	88.3 (98)	1.15	0.56, 2.34	
	Routine visit	62	14.4	11.3 (7)	88.7 (55)	1.10	0.45, 2.68	
	Not applicable	241	55.8	10.4 (25)	89.6 (216)	*Ref*		
	Unknown	2	0.5	0.0 (0)	100.0 (2)	NA		
Patient hospitalised	For the day only	32	7.4	6.3 (2)	93.8 (30)	0.60	0.13, 2.57	0.26
	For longer than 24 hours	3	0.7	33.3 (1)	66.7 (2)	4.34	0.38, 49.59	
	No	153	35.4	15.0 (23)	85.0 (130)	1.54	0.84, 2.82	

	Not applicable	242	56.0	10.3 (25)	89.7 (217)	*Ref*		
	Unknown	2	0.5	0.0 (0)	100.0 (2)	NA		
5. Preventative healthcare and exposure to other animals and carehomes								
Antiparasite treatment given	No treatment (yes)	69	16.0	23.2 (16)	76.8 (53)	2.83	1.46, 5.47	0.00
	No treatment (no)	363	84.0	9.4 (35)	90.4 (328)	*Ref*		
	Vet prescribed treatment (yes)	264	61.1	6.9 (18)	93.1 (246)	0.30	0.16, 0.55	<0.001
	Vet prescribed treatment (no)	168	38.9	19.6 (33)	80.4 (135)	*Ref*		
	Over the counter/shop bought treatment (yes)	41	9.5	12.2 (5)	87.8 (36)	1.04	0.39, 2.79	0.94
	Over the counter/shop bought treatment (no)	391	90.5	11.8 (46)	88.2 (345)	*Ref*		
	Natural remedy (yes)	72	16.7	18.1 (13)	81.9 (59)	1.87	0.94, 3.72	0.08
	Natural remedy (no)	360	83.3	10.6 (38)	89.4 (322)	*Ref*		
Regular contact with other animals	Dogs (yes)	380	88.0	12.1 (46)	87.9 (334)	1.30	0.49, 3.42	0.60
	Dogs (no)	52	12.0	9.6 (5)	90.4 (47)	*Ref*		
	Cats (yes)	149	34.5	9.4 (14)	90.6 (135)	0.69	0.36, 1.32	0.26
	Cats (no)	283	65.5	13.1 (37)	86.9 (246)	*Ref*		
	Small mammals/rodents (yes)	56	13.0	8.9 (5)	91.1 (51)	0.70	0.27, 1.85	0.48
	Small mammals/rodents (no)	376	87.0	12.2 (46)	87.8 (330)	*Ref*		
	Horses (yes)	81	18.8	9.9 (8)	90.1 (73)	0.79	0.35, 1.74	0.55
	Horses (no)	351	81.3	12.3 (43)	87.7 (308)	*Ref*		
	Farm animals (yes)	75	17.4	9.3 (7)	90.7 (68)	0.73	0.32, 1.70	0.47
	Farm animals (no)	357	82.6	12.3 (44)	87.7 (313)	*Ref*		
	Wildlife (yes)	126	29.2	11.1 (14)	88.9 (112)	0.91	0.47, 1.75	0.77
	Wildlife (no)	306	70.8	12.1 (37)	87.9 (269)	*Ref*		
	Reptiles/snakes (yes)	13	3.0	15.4 (2)	84.6 (11)	1.37	0.30, 6.38	0.69
	Reptiles/snakes (no)	419	97.0	11.7 (49)	88.3 (370)	*Ref*		
	Chickens/poultry (yes)	29	6.7	10.3 (3)	89.7 (26)	0.85	0.25, 2.93	0.80
	Chickens/poultry (no)	403	93.3	11.9 (48)	88.1 (355)	*Ref*		
	No other regular contact (yes)	26	6.0	7.7 (2)	92.3 (24)	0.97	0.28, 3.36	0.97
	No other regular contact (no)	406	94.0	11.8 (48)	88.2 (358)	*Ref*		
	Other (yes)	41	9.5	12.2 (5)	87.8 (36)	1.04	0.39, 2.79	0.94

	Other (no)		391	90.5	11.8 (46)	88.2 (345)	*Ref*		
Regular access to communal areas	Dog training classes (yes)		77	17.8	16.9 (13)	83.1 (64)	1.69	0.85, 3.36	0.13
	Dog training classes (no)		355	82.2	10.7 (38)	89.3 (317)	*Ref*		
	Doggy daycare (yes)		26	6.0	23.1 (6)	76.9 (20)	2.41	0.92, 6.31	0.07
	Doggy daycare (no)		406	94.0	11.1 (45)	88.9 (361)	*Ref*		
	Group dog walking (yes)		67	15.5	17.9 (12)	82.1 (55)	1.82	0.90, 3.70	0.10
	Group dog walking (no)		365	84.5	10.7 (39)	89.3 (326)	*Ref*		
	Dog shows (yes)		40	9.3	22.5 (9)	77.5 (31)	2.41	1.08, 5.41	0.03
	Dog shows (no)		391	90.7	10.7 (42)	89.3 (349)	*Ref*		
	Dog parks (yes)		77	17.8	10.4 (8)	89.6 (69)	0.84	0.38, 1.87	0.67
	Dog parks (no)		355	82.2	12.1 (43)	87.9 (312)	*Ref*		
	Farm land (yes)		222	51.4	10.8 (24)	89.2 (198)	0.82	0.46, 1.48	0.51
	Farm land (no)		210	48.6	12.9 (27)	87.1 (183)	*Ref*		
	Public parks/towpaths/footpaths (yes)		354	81.9	11.6 (41)	88.4 (313)	0.89	0.43, 1.87	0.76
	Public parks/towpaths/footpaths (no)		78	18.1	12.8 (10)	87.2 (68)	*Ref*		
	Other (yes)		70	16.2	7.1 (5)	92.9 (65)	0.53	0.20, 1.38	0.19
	Other (no)		362	83.8	12.7 (46)	87.3 (316)	*Ref*		
	No regular access to places listed (yes)		18	4.2	5.6 (1)	94.4 (17)	0.43	0.06, 3.29	0.42
	No regular access to places listed (no)		414	95.8	12.1 (50)	87.9 (364)	*Ref*		
Visit human carehomes (e.g. PAT dog)	Yes		8	1.9	37.5 (3)	62.5 (5)	4.66	1.80, 20.13	0.04
	No		421	97.5	11.4 (48)	88.6 (373)	*Ref*		
	Unknown		3	0.7	0.0 (0)	100.0 (3)	NA		
6. Household data									
Number of people in household		1	66	15.3	13.6 (9)	86.4 (57)	*Ref*		0.91
		2	220	50.9	11.4 (25)	88.6 (195)	0.81	0.36, 1.84	
		3	69	16.0	10.1 (7)	89.9 (62)	0.72	0.25, 1.05	
		4	58	13.4	12.1 (7)	87.9 (51)	0.87	0.30, 2.50	
	5 or more		17	3.9	17.6 (3)	82.4 (14)	1.36	0.32, 5.68	
	Unknown		2	0.5	0.0 (0)	100.0 (2)	NA		
Residents present aged 65 or over	Yes		87	20.1	16.1 (14)	83.9 (73)	1.58	0.81, 3.08	0.18

	No	382	79.2	9.7 (37)	79.8 (305)	*Ref*		
	Unknown	3	0.7	0.0 (0)	100.0 (3)	NA		
Residents present aged 5 or younger	Yes	27	6.3	7.4 (2)	92.6 (25)	0.58	0.13, 2.51	0.46
	No	402	93.1	12.2 (49)	87.8 (353)	*Ref*		
	Unknown	3	0.7	0.0 (0)	100.0 (3)	NA		
Resident works in riskier areas	Hospital/GP surgery (yes)	27	6.3	18.5 (5)	81.5 (22)	1.77	0.64, 4.91	0.27
	Hospital/GP surgery (no)	405	93.8	11.4 (46)	88.6 (359)	*Ref*		
	Carehome (yes)	8	1.9	12.5 (1)	87.5 (7)	1.07	0.13, 8.87	0.95
	Carehome (no)	424	98.1	11.8 (50)	88.2 (374)	*Ref*		
	Nursery (yes)	3	0.7	66.7 (2)	33.3 (1)	15.51	1.38, 174.2	0.03
	Nursery (no)	429	99.3	11.4 (49)	88.6 (380)	*Ref*		
	Primary school (yes)	11	2.5	9.1 (1)	90.9 (10)	0.74	0.09, 5.92	0.78
	Primary school (no)	421	97.5	11.9 (50)	88.1 (371)	*Ref*		
-	Livestock farm (yes)	13	3.0	15.4 (2)	84.6 (11)	1.37	0.30, 6.38	0.69
	Livestock farm (no)	419	97.0	11.7 (49)	88.3 (370)	*Ref*		
	Dog boarding kennels (yes)	9	2.1	33.3 (3)	66.7 (6)	3.91	0.95, 16.13	0.06
	Dog boarding kennels (no)	423	97.9	11.3 (48)	88.7 (375)	*Ref*		
	Petting zoo (yes)	2	0.5	0.0 (0)	100.0 (2)			1.00
	Petting zoo (no)	430	99.5	11.9 (51)	88.1 (379)	*Ref*		
	Veterinary practice (yes)	113	26.2	5.3 (6)	94.7 (107)	0.34	0.14, 0.82	0.02
	Veterinary practice (no)	319	73.8	14.1 (45)	85.9 (274)	*Ref*		
	No other risky workplace (yes)	259	60.0	13.1 (34)	86.9 (225)	1.39	0.75, 2.57	0.30
	No other risky workplace (no)	173	40.0	9.8 (17)	90.2 (156)	*Ref*		
Resident received antibiotics last 3 months	Yes	51	11.8	11.8 (6)	88.2 (45)	0.98	0.40, 2.44	0.97
	No	377	87.3	11.9 (45)	88.1 (332)	*Ref*		
	Unknown	4	0.9	0.0 (0)	100.0 (4)	NA		
Resident hospitalised in last 3 months	Yes	15	3.5	13.3 (2)	86.7 (13)	1.14	0.25, 5.20	0.87
	No	412	95.4	11.9 (49)	88.1 (363)	*Ref*		
	Unknown	5	1.2	0.0 (0)	100.0 (5)	NA		

Region of country	East Midlands	28	6.5	3.6 (1)	96.4 (27)	0.7	0.06, 8.16	0.26
	East of England	27	6.3	14.8 (4)	85.2 (23)	3.3	0.56, 19.49	
	Greater London	12	2.8	16.7 (2)	83.3 (10)	3.8	0.48, 30.42	
	North East and Yorkshire	40	9.3	5.0 (2)	95.0 (38)	*Ref*		
	North West	104	24.1	8.7 (9)	91.3 (95)	1.8	0.37, 8.72	
	Wales, Scotland and Northern Ireland	54	12.8	9.3 (5)	90.7 (49)	1.94	0.36, 10.55	
	South East	73	16.9	16.4 (12)	83.6 (61)	3.74	0.79, 17.62	
	South West	47	10.9	21.3 (10)	78.7 (37)	5.14	1.05, 25.04	
	West Midlands	36	8.3	11.1 (4)	88.9 (32)	2.38	0.41, 13.82	
	Unknown	11	2.5	18.2 (2)	81.8 (9)	NA		
7. Dog data								
Dog sex	Female entire	48	11.1	20.8 (10)	79.2 (38)	2.8	1.15, 6.80	0.15
	Female neutered	163	37.7	8.6 (14)	91.4 (149)	*Ref*		
	Male entire	68	15.7	13.2 (9)	86.8 (59)	1.62	0.67, 3.95	
	Male neutered	150	34.7	12.0 (18)	88.0 (132)	1.45	0.70, 3.03	
	Unknown	3	0.7	0.0 (0)	100.0 (3)	NA		
Dog age	<12 months	29	6.7	13.8 (4)	86.2 (25)	0.96	0.88, 1.03	0.26
	1 year	16	3.7	6.3 (1)	93.8 (15)			
	2 years	42	9.7	16.7 (7)	83.3 (35)			
	3 years	38	8.8	15.8 (6)	84.2 (32)			
	4 years	36	8.3	8.3 (3)	91.7 (33)			
	5 years	37	8.6	10.8 (4)	89.2 (33)			
	6 years	36	8.3	22.2 (8)	77.8 (28)			
	7 years	34	7.9	11.8 (4)	88.2 (30)			
	8 years or older	157	36.9	8.9 (14)	91.1 (143)			
	Unknown	7	1.6	0.0 (0)	100.0 (7)	NA		
Length of time owned	<12 months	44	10.4	13.6 (6)	86.4 (38)	0.96	0.88, 1.05	0.35
	1 year	23	5.4	8.7 (2)	91.3 (21)			

	2 years	49	11.5	12.2 (6)	87.8 (43)			
	3 years	43	10.1	16.3 (7)	83.7 (43)			
	4 years	57	13.4	14.0 (8)	86.0 (49)			
	5 years	36	8.5	16.7 (6)	83.3 (30)			
	6 years	31	7.3	12.9 (4)	87.1 (27)			
	7 years	28	6.6	7.1 (2)	92.9 (26)			
	8 years or longer	114	26.8	8.8 (10)	91.2 (104)			
	Unknown	7	1.6	0.0 (0)	100.0 (7)	NA		

Table A4: *Univariable analysis of explanatory factors associated with dog faecal carriage of multidrug resistant (MDR)-E. coli, analysed at sample level (N=432 dogs). Ref=reference category*

**Variable**	**Category**	**N (total samples)**	**% of total**	**MDR-*E. coli* present % (N)**		**Odds ratio**	**95% CI**	**p value**
				Yes	***No***			
		**432**		**8.1 (35)**	**91.9 (397)**			
1. Food								
Fed raw diet	Yes	193	55.3	18.1 (35)	81.9 (158)	17.43	5.27, 57.64	<0.001
	No	239	44.7	1.3 (3)	98.7 (236)	*Ref*		
Type of diet fed	Pre-prepared raw yes	157	36.3	17.8 (28)	82.2 (129)	5.75	2.71, 12.20	<0.001
	Pre-prepared raw no	275	63.7	3.6 (10)	96.4 (265)	*Ref*		
	DIY/home-prepared raw yes	101	23.4	15.8 (16)	84.2 (85)	2.64	1.33, 5.26	0.01
	DIY/home-prepared raw no	331	76.6	6.6 (22)	93.4 (309)	*Ref*		
	Cooked meat/bones yes	43	10.0	4.7 (2)	95.3 (41)	0.48	0.11, 2.06	0.32
	Cooked meat/bones no	389	90.0	9.3 (36)	90.7 (353)	*Ref*		
	Cooked commercial complete dry/kibble yes	254	58.8	2.4 (6)	97.6 (248)	0.11	0.05, 0.27	<0.001

	Cooked commercial complete dry/kibble no	178	41.2	18.0 (32)	82.0 (146)	*Ref*		
	Cooked commercial complete wet yes	108	25.0	1.9 (2)	98.1 (106)	0.15	0.04, 0.64	0.02
	Cooked commercial complete wet no	324	75.0	11.1 (36)	88.9 (288)	*Ref*		
	Vegetarian/vegan yes	4	0.9	25.0 (1)	75.0 (3)	3.52	0.36, 34.72	0.28
	Vegetarian/vegan no	428	99.1	8.6 (37)	91.4 (391)	*Ref*		
	Other yes	39	9.0	5.1 (2)	94.9 (37)	0.54	0.12, 2.32	0.4
	Other no	393	91.0	9.2 (36)	90.8 (357)	*Ref*		
Diet changed in last 3 months	Yes	83	19.2	1.2 (1)	98.8 (82)	0.21	0.05, 0.91	0.04
	No	347	80.3	9.8 (34)	90.2 (313)	*Ref*		
	Unknown	2	0.5	0.0 (0)	100.0 (2)	NA		
Types of treat fed	Shop bought cooked treats/biscuits yes	273	63.2	4.4 (12)	95.6 (261)	0.24	0.12, 0.48	<0.001
	Shop bought cooked treats/biscuits no	159	36.8	16.4 (26)	83.6 (133)	*Ref*		
	Freeze dried treats yes	136	31.5	12.5 (17)	87.5 (119)	1.87	0.95, 3.67	0.07
	Freeze dried treats no	296	68.5	7.1 (21)	92.9 (275)	*Ref*		
	Dried treats yes	159	36.8	12.6 (20)	87.4 (139)	2.04	1.04, 3.98	0.04
	Dried treats no	273	63.2	6.6 (18)	93.4 (255)	*Ref*		
	Raw meat yes	75	17.4	16.0 (12)	84.0 (63)	2.43	1.16, 5.06	0.02
	Raw meat no	357	82.6	7.3 (26)	92.7 (331)	*Ref*		
	Raw bones yes	97	22.5	15.5 (15)	84.5 (82)	2.48	1.24, 4.97	0.01
	Raw bones no	335	77.5	6.9 (23)	93.1 (312)	*Ref*		

	Cooked meat yes	104	24.1	4.8 (5)	95.2 (99)	0.45	0.17, 1.19	0.11
	Cooked meat no	328	75.9	10.1 (33)	89.9 (295)	*Ref*		
	Cooked bones yes	10	2.3	0.0 (0)	100.0 (10)			1.00
	Cooked bones no	422	97.7	8.3 (35)	91.7 (387)			
	I don't feed any treats yes	16	3.7	12.5 (2)	87.5 (14)	1.51	0.33, 6.90	0.6
	I don't feed any treats no	416	96.3	7.9 (33)	92.1 (383)	*Ref*		
	Other treats yes	110	25.5	11.8 (13)	88.2 (97)	1.59	0.78, 3.32	0.2
	Other treats no	322	74.5	7.8 (25)	92.2 (297)	*Ref*		
Human food/titbits given	Frequently	70	16.2	4.3 (3)	95.7 (67)	0.47	0.12, 1.91	0.04
	Occasionally as a treat	169	39.1	6.5 (11)	93.5 (158)	0.74	0.27, 1.98	
	Rarely	109	25.2	15.6 (17)	84.4 (92)	1.95	0.77, 4.96	
	No	81	18.8	8.6 (7)	91.4 (74)	*Ref*		
	Unknown	3	0.7	33.3 (1)	66.7 (2)	NA		
Dog scavenges	Yes frequently	74	17.1	4.1 (3)	95.9 (71)	0.46	0.13, 1.62	0.19
	Yes sometimes	165	38.2	11.5 (19)	88.5 (146)	1.41	0.70, 2.84	
	No	189	43.8	8.5 (16)	91.5 (173)	*Ref*		
	Unknown	4	0.9	0.0 (0)	100.0 (4)	NA		
2. Antibiotic use								
Antibiotics in last 3 months	Yes	47	10.9	17.0 (8)	83.0 (39)	2.43	1.04, 5.66	0.04
	No	385	89.1	7.8 (30)	92.2 (355)	*Ref*		

Currently receiving antibiotics	Yes	5	1.2	20.0 (1)	80.0 (4)	2.71	0.30, 24.88	0.38
	No	426	98.6	8.5 (36)	91.5 (390)	*Ref*		
	Unknown	1	0.2	0.0 (0)	100.0 (1)	NA		
Most recent antibiotic course	1 week ago or less	4	0.9	0.0 (0)	100.0 (4)			0.39
	2-8 weeks ago	17	3.9	17.6 (3)	82.4 (14)			
	More than 8 weeks ago	22	5.1	13.6 (3)	86.4 (19)			
	Not applicable	388	89.8	8.0 (31)	92.0 (357)	*Ref*		
	Unknown	1	0.2	0.0 (0)	100.0 (1)	NA		
Duration of most recent course	One off injection	3	0.7	33.3 (1)	66.7 (2)	5.9	0.52, 66.97	0.08
	Oral antibiotics up to 5 days	16	3.7	6.3 (1)	93.8 (15)	0.79	0.10, 6.16	
	Oral antibiotics up to 10 days	20	4.6	25.0 (5)	75.0 (15)	3.93	1.34, 11.57	
	Oral antibiotics for 2 weeks or longer	7	1.6	14.3 (1)	85.7 (6)	1.97	0.23, 16.88	
	Not applicable	384	88.9	7.8 (30)	92.2 (354)	*Ref*		
	Unknown	2	0.5	0.0 (0)	100.0 (2)	NA		
3. Diarrhoea								
Diarrhoea/loose stools last 3 months	Yes	138	31.9	6.5 (9)	93.5 (129)	0.64	0.29, 1.38	0.25
	No	293	67.8	9.9 (29)	90.1 (264)	*Ref*		
	Unknown	1	0.2	0.0 (0)	100.0 (1)	NA		
Most recent episode	Currently has/always has/in the last week	18	4.2	5.6 (1)	94.4 (17)			0.57
	1-2 weeks ago	35	8.1	11.4 (4)	88.6 (31)			

	2-4 weeks ago	36	8.3	8.3 (3)	91.7 (33)			
	4-8 weeks ago	28	6.5	0.0 (0)	100.0 (28)			
	More than 8 weeks ago	21	4.9	4.8 (1)	95.2 (20)			
	Not applicable	292	67.6	9.9 (29)	90.1 (263)			
	Unknown	2	0.5	0.0 (0)	100.0 (2)			
Most recent episode (categories combined so no zeros)	Currently has/always has/in the last week	18	4.2	5.6 (1)	94.4 (17)	0.53	0.07, 4.15	0.53
	1-2 weeks ago	35	8.1	11.4 (4)	88.6 (31)	1.17	0.39, 3.55	
	2-4 weeks ago	36	8.3	8.3 (3)	91.7 (33)	0.82	0.24, 2.86	
	More than 4 weeks ago	49	11.3	2.0 (1)	98.0 (48)	0.19	0.03, 1.42	
	Not applicable	292	67.6	9.9 (29)	90.1 (263)	*Ref*		
	Unknown	2	0.5	0.0 (0)	100.0 (2)	NA		
Repeated episodes in last 3 months	Yes	46	10.6	4.2 (2)	95.7 (44)	0.41	0.10, 1.80	0.44
	No	91	21.1	7.7 (7)	92.3 (84)	0.76	0.32, 1.80	
	Not applicable	293	67.8	9.9 (29)	90.1 (264)	*Ref*		
	Unknown	2	0.5	0.0 (0)	100.0 (2)	NA		
Number of episodes in last 3 months (combined)	Constant diarrhoea/up to 2 episodes	26	6.0	3.8 (1)	96.2 (25)	0.39	0.05, 2.93	0.76
	3-4 episodes	15	3.5	6.7 (1)	93.3 (14)	0.69	0.09, 5.39	
	5 or more episodes	17	3.9	5.9 (1)	94.1 (16)	0.6	0.08, 4.68	
	Not applicable	372	86.1	9.4 (35)	90.6 (337)	*Ref*		
	Unknown	2	0.5	0.0 (0)	100.0 (2)	NA		

Treatment given	None, resolved by itself (yes)	71	16.4	5.6 (4)	94.4 (67)	0.57	0.20, 1.67	0.31
	None, resolved by itself (no)	361	83.6	9.4 (34)	90.6 (327)	*Ref*		
	Bland diet (yes)	25	5.8	4.0 (1)	96.0 (24)	0.42	0.06, 3.17	0.4
	Bland diet (no)	407	94.2	9.1 (37)	90.9 (370)	*Ref*		
	Home remedy (yes)	11	2.5	18.2 (2)	81.8 (9)	2.38	0.50, 11.42	0.28
	Home remedy (no)	421	97.5	8.6 (36)	91.4 (385)	*Ref*		
	Over the counter medication from a shop (yes)	25	5.8	0.0 (0)	100.0 (25)			0.15
	Over the counter medication from a shop (no)	407	94.2	9.3 (38)	90.7 (369)			
	Veterinary prescribed treatment (yes)	19	4.4	10.5 (2)	89.5 (17)	1.23	0.27, 5.55	0.790
	Veterinary prescribed treatment (no)	413	95.6	8.7 (36)	91.3 (377)	*Ref*		
4.Vet visits								
Visit to vet in the last 3 months	Yes	189	43.8	11.1 (21)	88.9 (168)	1.65	0.85, 3.23	0.14
	No	242	46.0	7.0 (17)	93.0 (225)			
	Unknown	1	0.2	0.0 (0)	100.0 (1)			
Number of vet visits	1	102	23.6	12.7 (13)	87.3 (89)	2.05	0.95, 4.45	0.57
	2	43	10.0	11.6 (5)	88.4 (38)	1.85	0.64, 5.35	
	3	22	5.1	9.1 (2)	90.9 (20)	1.41	0.30, 6.56	
	4	8	1.9	12.5 (1)	87.5 (7)	2.01	0.23, 17.35	
	5 or more visits	14	3.2	7.1 (1)	92.9 (13)	1.08	0.13, 8.80	
	Not applicable	241	55.8	6.6 (16)	93.4 (225)	*Ref*		

	Unknown	2	0.5	0.0 (0)	100.0 (2)	NA		
Reason for visit	Emergency	16	3.7	37.5 (6)	62.5 (10)	8.44	2.72, 26.17	0.003
	Non-emergency problem/concern	111	25.7	9.9 (11)	90.1 (100)	1.55	0.69, 3.45	
	Routine visit	62	14.4	8.1 (5)	91.9 (57)	1.23	0.43, 3.51	
	Not applicable	241	55.8	6.6 (16)	93.4 (225)	*Ref*		
	Unknown	2	0.5	0.0 (0)	100.0 (2)	NA		
Reason for visit<br>Categories collapsed	All (emergency/non-emergency/routine)	189	43.7	11.6 (22)	88.4 (167)			
	No visit/not applicable	241	55.8	6.6 (16)	93.4 (225)	*Ref*		
	Unknown	2	0.5	0.0 (0)	100.0 (2)	NA		
Patient hospitalised	For the day only	32	7.4	3.1 (1)	96.9 (31)	0.46	0.06, 3.56	0.06
	For longer than 24 hours	3	0.7	33.3 (1)	66.7 (2)	7.06	0.61, 82.12	
	No	153	35.4	13.1 (20)	86.9 (133)	2.12	1.06, 4.24	
	Not applicable	242	56.0	6.6 (16)	93.4 (226)	*Ref*		
	Unknown	2	0.5	0.0 (0)	100.0 (2)	NA		
5. Preventative healthcare and exposure to other animals and carehomes								
Antiparasite treatment given	No treatment (yes)	69	16.0	15.9 (11)	84.1 (58)	2.36	1.11, 5.02	0.03
	No treatment (no)	363	84.0	7.4 (27)	92.6 (336)	*Ref*		
	Vet prescribed treatment (yes)	264	61.1	5.3 (14)	94.7 (250)	0.34	0.17, 0.67	0.002
	Vet prescribed treatment (no)	168	38.9	14.3 (24)	85.7 (144)	*Ref*		
	Over the counter/shop bought treatment (yes)	41	9.5	7.3 (3)	92.7 (38)	0.8	0.24, 2.74	0.73

	Over the counter/shop bought treatment (no)	391	90.5	9.0 (35)	91.0 (356)	*Ref*		
	Natural remedy (yes)	72	16.7	13.9 (10)	86.1 (62)	1.91	0.88, 4.14	0.1
	Natural remedy (no)	360	83.3	7.8 (28)	92.2 (332)	*Ref*		
Regular contact with other animals	Dogs (yes)	380	88.0	7.7 (34)	92.3 (346)	1.18	0.40, 3.47	0.77
	Dogs (no)	52	12.0	7.7 (4)	92.3 (48)	*Ref*		
	Cats (yes)	149	34.5	7.4 (11)	92.6 (138)	0.76	0.36, 1.57	0.45
	Cats (no)	283	65.5	9.5 (27)	90.5 (256)	*Ref*		
	Small mammals/rodents (yes)	56	13.0	3.6 (2)	96.4 (54)	0.35	0.08, 1.40	0.16
	Small mammals/rodents (no)	376	87.0	9.6 (36)	90.4 (340)	*Ref*		
	Horses (yes)	81	18.8	2.5 (2)	97.5 (79)	0.22	0.52, 0.94	0.04
	Horses (no)	351	81.3	10.3 (36)	89.7 (315)	*Ref*		
	Farm animals (yes)	75	17.4	4.0 (3)	96.0 (72)	0.38	0.12, 1.28	0.12
	Farm animals (no)	357	82.6	9.8 (35)	90.2 (322)	*Ref*		
	Wildlife (yes)	126	29.2	7.1 (9)	92.9 (117)	0.74	0.34, 1.60	0.44
	Wildlife (no)	306	70.8	9.5 (29)	90.5 (277)	*Ref*		
	Reptiles/snakes (yes)	13	3.0	15.4 (2)	84.6 (11)	1.93	0.41, 9.07	0.40
	Reptiles/snakes (no)	419	97.0	8.6 (36)	91.4 (383)	*Ref*		
	Chickens/poultry (yes)	29	6.7	3.4 (1)	96.6 (28)	0.35	0.05, 2.67	0.31
	Chickens/poultry (no)	403	93.3	9.2 (37)	90.8 (366)	*Ref*		
	No other regular contact (yes)	26	6.0	7.7 (2)	92.3 (24)	0.86	0.19, 3.77	0.84

	No other regular contact (no)	406	94.0	8.9 (36)	91.1 (370)	*Ref*		
	Other (yes)	41	9.5	4.9 (2)	95.1 (39)	0.51	0.12, 2.18	0.36
	Other (no)	391	90.5	9.2 (36)	90.8 (355)	*Ref*		
Regular access to communal areas	Dog training classes (yes)	77	17.8	14.3 (11)	85.7 (66)	2.03	0.96, 4.28	0.07
	Dog training classes (no)	355	82.2	7.6 (27)	92.4 (328)	*Ref*		
	Doggy daycare (yes)	26	6.0	19.2 (5)	80.8 (21)	2.69	0.95, 7.60	0.06
	Doggy daycare (no)	406	94.0	8.1 (33)	91.9 (373)	*Ref*		
	Group dog walking (yes)	67	15.5	13.4 (9)	86.6 (58)	1.8	0.81, 3.99	0.15
	Group dog walking (no)	365	84.5	7.9 (29)	92.1 (336)	*Ref*		
	Dog shows (yes)	40	9.3	17.5 (7)	82.5 (33)	2.46	1.01, 6.02	0.05
	Dog shows (no)	391	90.7	7.9 (31)	92.1 (360)	*Ref*		
	Dog parks (yes)	77	17.8	6.5 (5)	93.5 (72)	0.68	0.26, 1.80	0.43
	Dog parks (no)	355	82.2	9.3 (33)	90.7 (322)	*Ref*		
	Farm land (yes)	222	51.4	6.3 (14)	93.7 (208)	0.52	0.26, 1.04	0.06
	Farm land (no)	210	48.6	11.4 (24)	88.6 (186)	*Ref*		
	Public parks/towpaths/footpaths (yes)	354	81.9	9.0 (32)	91.0 (322)	1.19	0.48, 2.96	0.7
	Public parks/towpaths/footpaths (no)	78	18.1	7.7 (6)	92.3 (72)	*Ref*		
	Other (yes)	70	16.2	5.7 (4)	94.3 (66)	0.59	0.20, 1.70	0.33
	Other (no)	362	83.8	9.4 (34)	90.6 (328)	*Ref*		
	No regular access to places listed (yes)	18	4.2	5.6 (1)	94.4 (17)	0.6	0.08, 4.63	0.62

	No regular access to places listed (no)	414	95.8	8.9 (37)	91.1 (377)	*Ref*		
Visit human carehomes (e.g. PAT dog)	Yes	8	1.9	25.0 (2)	75.0 (6)	3.57	0.69, 18.31	0.13
	No	421	97.5	8.6 (36)	91.4 (385)	*Ref*		
	Unknown	3	0.7	0.0 (0)	100.0 (3)	NA		
6. Household data								
Number of people in household	1	66	15.3	10.6 (7)	89.4 (59)	*Ref*		0.83
	2	220	50.9	8.2 (18)	91.8 (202)	0.75	0.30, 1.89	
	3	69	16.0	7.2 (5)	92.8 (64)	0.66	0.20, 2.19	
	4	58	13.4	12.1 (7)	87.9 (51)	1.16	0.38, 3.52	
	5 or more	17	3.9	5.9 (1)	95.1 (16)	0.53	0.06, 4.60	
	Unknown	2	0.5	0.0 (0)	100.0 (2)	NA		
Residents present aged 65 or over?	Yes	87	20.1	11.5 (10)	88.5 (77)	1.46	0.68, 3.13	0.34
	No	382	79.2	8.2 (28)	91.8 (314)	*Ref*		
	Unknown	3	0.7	0.0 (0)	100.0 (3)	NA		
Residents present aged 5 or younger?	Yes	27	6.3	7.4 (2)	92.6 (25)	0.81	0.19, 3.57	0.78
	No	402	93.1	9.0 (36)	91.0 (366)	*Ref*		
	Unknown	3	0.7	0.0 (0)	100.0 (3)	NA		
Resident works in riskier areas	Hospital/GP surgery (yes)	27	6.3	11.1 (3)	88.9 (24)	1.32	0.38, 4.61	0.66
	Hospital/GP surgery (no)	405	93.8	8.6 (35)	91.4 (370)	*Ref*		
	Carehome (yes)	8	1.9	12.5 (1)	87.5 (7)	1.5	0.18, 12.48	0.71

	Carehome (no)	424	98.1	8.7 (37)	91.3 (387)	*Ref*		
	Nursery (yes)	3	0.7	66.7 (2)	33.3 (1)	21.8	1.93, 246.67	0.01
	Nursery (no)	429	99.3	8.4 (36)	91.6 (393)	*Ref*		
	Primary school (yes)	11	2.5	9.1 (1)	90.9 (10)	1.04	0.13, 8.33	0.97
	Primary school (no)	421	97.5	8.8 (37)	91.2 (384)	*Ref*		
-	Livestock farm (yes)	13	3.0	7.7 (1)	92.3 (12)	0.86	0.11, 6.80	0.88
	Livestock farm (no)	419	97.0	8.8 (37)	91.2 (382)	*Ref*		
	Dog boarding kennels (yes)	9	2.1	33.3 (3)	66.7 (6)	5.54	1.32, 23.13	0.02
	Dog boarding kennels (no)	423	97.9	8.3 (35)	91.7 (388)	*Ref*		
	Petting zoo (yes)	2	0.5	0.0 (0)	100.0 (2)			1.0
	Petting zoo (no)	430	99.5	8.8 (38)	91.2 (392)	*Ref*		
	Veterinary practice (yes)	113	26.2	3.5 (4)	96.5 (109)	0.31	0.11, 0.89	0.03
	Veterinary practice (no)	319	73.8	10.7 (34)	89.3 (285)	*Ref*		
	No other risky workplace (yes)	259	60.0	10.0 (26)	90.0 (233)	1.5	0.73, 3.05	0.27
	No other risky workplace (no)	173	40.0	6.9 (12)	93.1 (161)	*Ref*		
Resident received antibiotics last 3 months	Yes	51	11.8	9.8 (5)	90.2 (46)	1.13	0.42, 3.05	0.81
	No	377	87.3	8.8 (33)	91.2 (344)	*Ref*		
	Unknown	4	0.9	0.0 (0)	100.0 (4)	NA		
Resident hospitalised in last 3 months	Yes	15	3.5	13.3 (2)	86.7 (13)	1.61	0.35, 7.40	0.54
	No	412	95.4	8.7 (36)	91.3 (376)	*Ref*		

	Unknown	5	1.2	0.0 (0)	100.0 (5)	NA		
Region of country (combined)	East Midlands	28	6.5	3.6 (1)	96.4 (27)	0.70	0.06, 8.16	0.78
NE as ref	East of England	27	6.3	7.4 (2)	92.6 (25)	1.52	0.20, 11.50	
	Greater London	12	2.8	16.7 (2)	83.3 (10)	3.80	0.48, 30.42	
	North East and Yorkshire	40	9.3	5.0 (2)	95.0 (38)	*Ref*		
	North West	104	24.1	7.7 (8)	92.3 (96)	1.58	0.32, 7.80	
	Wales, Scotland and Northern Ireland	54	12.8	9.3 (5)	90.7 (49)	1.94	0.36, 10.55	
	South East	73	16.9	9.6 (7)	90.4 (66)	2.02	0.40, 10.20	
	South West	47	10.9	14.9 (7)	85.1 (40)	3.33	0.65, 17.02	
	West Midlands	36	8.3	8.3 (3)	91.7 (33)	1.73	0.27, 10.97	
	Unknown	11	2.5	9.1 (1)	90.9 (10)	NA		
Region of country (combined)	East Midlands	28	6.5	3.6 (1)	96.4 (27)	0.44	0.05, 3.71	0.78
NW as ref	East of England	27	6.3	7.4 (2)	92.6 (25)	0.96	0.19, 4.81	
	Greater London	12	2.8	16.7 (2)	83.3 (10)	2.4	0.45, 12.89	
	North East and Yorkshire	40	9.3	5.0 (2)	95.0 (38)	0.63	0.13, 3.11	
	North West	104	24.1	7.7 (8)	92.3 (96)	*Ref*		
	Wales, Scotland and Northern Ireland	54	12.8	9.3 (5)	90.7 (49)	1.22	0.38, 3.94	
	South East	73	16.9	9.6 (7)	90.4 (66)	1.27	0.44, 3.68	
	South West	47	10.9	14.9 (7)	85.1 (40)	2.1	0.71, 6.18	
	West Midlands	36	8.3	8.3 (3)	91.7 (33)	1.09	0.27, 4.36	

	Unknown	11	2.5	9.1 (1)	90.9 (10)	NA		
7. Dog data								
Dog sex	Female entire	48	11.1	10.7 (6)	87.5 (42)	2.19	0.75, 6.36	0.29
	Female neutered	163	37.7	6.1 (10)	93.9 (153)	*Ref*		
	Male entire	68	15.7	13.2 (9)	86.8 (59)	2.33	0.90, 6.03	
	Male neutered	150	34.7	8.7 (13)	91.3 (137)	1.45	0.62, 3.42	
	Unknown	3	0.7	0.0 (0)	100.0 (3)	NA		
Dog age	<12 months	29	6.7	10.3 (3)	89.7 (26)	*Ref*		0.08
	1 year	16	3.7	6.3 (1)	93.8 (15)	0.58	0.06, 6.06	
	2 years	42	9.7	16.7 (7)	83.3 (35)	1.73	0.41, 7.35	
	3 years	38	8.8	15.8 (6)	84.2 (32)	1.63	0.37, 7.13	
	4 years	36	8.3	8.3 (3)	91.7 (33)	0.79	0.15, 4.23	
	5 years	37	8.6	10.8 (4)	89.2 (33)	1.05	0.22, 5.11	
	6 years	36	8.3	2.8 (1)	97.2 (35)	0.25	0.02, 2.52	
	7 years	34	7.9	8.8 (3)	91.2 (31)	0.84	0.16, 4.51	
	8 years or older	157	36.9	6.4 (10)	93.6 (147)	0.59	0.15, 2.29	
	Unknown	7	1.6	0.0 (0)	100.0 (7)	NA		
Length of time owned	<12 months	44	10.4	11.4 (5)	88.6 (39)	*Ref*		0.12
	1 year	23	5.4	8.7 (2)	91.3 (21)	0.74	0.13, 4.16	
	2 years	49	11.5	12.2 (6)	87.8 (43)	1.09	0.31, 3.85	

	3 years	43	10.1	16.3 (7)	83.7 (36)	1.52	0.44, 5.21	
	4 years	57	13.4	8.8 (5)	91.2 (52)	0.75	0.20, 2.77	
	5 years	36	8.5	8.3 (3)	91.7 (33)	0.71	0.16, 3.19	
	6 years	31	7.3	6.5 (2)	93.5 (29)	0.54	0.10, 2.97	
	7 years or longer	142	32.9	5.6 (8)	94.4 (134)	0.51	0.15, 1.70	
	Unknown	7	1.6	0.0 (0)	100.0 (7)	NA		

Table A5*: Types of antibiotics prescribed to dogs in the present study as recalled by dog owners, and the number (N) and percentage (%) of faecal samples (N=432) with 3GCR-, ESBL-producing and MDR-E. coli present*

**Variable**	**Category**	**N (total samples)**	**% of total**	**3GCR-*E. coli* present % (N)**		**ESBL-*E. coli* present % (N)**		**MDR-*E. coli* present % (N)**	
				Yes	***No***	***Yes***	***No***	***Yes***	***No***
		**432**		**17.4 (75)**	**82.6 (357)**	**11.8 (51)**	**88.2 (381)**	**8.1 (35)**	**91.9 (397)**
Antibiotic type	Amoxycillin-clavulanate	19	4.4	9.3 (7)	3.4 (12)	7.8 (4)	3.9 (15)	8.6 (3)	4.0 (16)
	Cefalexin	5	1.2	2.7 (2)	0.8 (3)	2.0 (1)	1.1 (4)	0.0 (0)	1.3 (5)
	Marbofloxacin	1	0.2	0.0 (0)	0.3 (1)	0.0 (0)	0.3 (1)	0.0 (0)	0.3 (1)
	Metronidazole	7	1.6	0.0 (0)	2.0 (7)	0.0 (0)	1.8 (7)	0.0 (0)	1.8 (7)
	Type not known	1	0.2	0.0 (0)	0.3 (1)	0.0 (0)	0.3 (1)	0.0 (0)	0.3 (1)
	Not applicable/ None prescribed	385	89.1	81.3 (61)	90.8 (324)	80.4 (41)	90.3 (344)	80.0 (28)	89.9 (357)
	Unknown	14	3.2	6.7 (5)	2.5 (9)	9.8 (5)	2.4 (9)	14.3 (5)	2.3 (9)
